# Ionic multiresonant thermally activated delayed fluorescence emitters for light emitting electrochemical cells

**DOI:** 10.3762/bjoc.18.136

**Published:** 2022-09-22

**Authors:** Merve Karaman, Abhishek Kumar Gupta, Subeesh Madayanad Suresh, Tomas Matulaitis, Lorenzo Mardegan, Daniel Tordera, Henk J Bolink, Sen Wu, Stuart Warriner, Ifor D Samuel, Eli Zysman-Colman

**Affiliations:** 1 Department of Material Science and Engineering, Faculty of Engineering and Architecture, Izmir Katip, Celebi University, Cigli, 35620-Izmir, Turkey; 2 Organic Semiconductor Centre, EaStCHEM School of Chemistry, University of St Andrews, St Andrews, UK, KY16 9ST; 3 Organic Semiconductor Centre, SUPA School of Physics and Astronomy, University of St Andrews, St Andrews KY16 9SS, UK; 4 Instituto de Ciencia Molecular (ICMol), Universidad de Valencia, C/Catedrático J. Beltrán 2, 46980 Paterna (Valencia), Spain; 5 School of Chemistry, University of Leeds, Woodhouse Lane, Leeds, UK

**Keywords:** electroluminescence, light-emitting electrochemical cells, multiresonance, purely organic emitters, thermally activated delayed fluorescence

## Abstract

We designed and synthesized two new ionic thermally activated delayed fluorescent (TADF) emitters that are charged analogues of a known multiresonant TADF (MR-TADF) compound, **DiKTa**. The emission of the charged derivatives is red-shifted compared to the parent compound. For instance, **DiKTa-OBuIm** emits in the green (λ_PL_ = 499 nm, 1 wt % in mCP) while **DiKTa-DPA-OBuIm** emits in the red (λ_PL_ = 577 nm, 1 wt % in mCP). In 1 wt % mCP films, both emitters showed good photoluminescence quantum yields of 71% and 61%, and delayed lifetimes of 316.6 μs and 241.7 μs, respectively, for **DiKTa-OBuIm** and **DiKTa-DPA-OBuIm**, leading to reverse intersystem crossing rates of 2.85 × 10^3^ s^−1^ and 3.04 × 10^3^ s^−1^. Light-emitting electrochemical cells were prepared using both **DiKTa-OBuIm** and **DiKTa-DPA-OBuIm** as active emitters showing green (λ_max_ = 534 nm) and red (λ_max_ = 656 nm) emission, respectively.

## Introduction

Light-emitting electrochemical cells (LEECs) are thin film light-emitting devices typically consisting of an emissive layer containing ionic species that facilitate charge transport and an emissive semiconductor material. The emissive layer is sandwiched between two air-stable electrodes [1]. Upon application of an external bias the ions in the active layer migrate to the corresponding electrodes, resulting in the formation of electrical double layers (EDLs) at the interface of the electrodes. The EDLs facilitate charge injection into the emissive layer regardless of the energy levels of the electroactive species and work function of the electrodes. Injection of electrons and holes creates oxidized and reduced species near the anode and cathode, respectively. These oxidized and reduced species are stabilized by the ions to form a p-i-n junction in the bulk of the emissive layer and emission takes place within the intrinsic region [2–6].

Two families of widely investigated emitters for LEECs are ionic transition metal complexes (iTMCs) [7–10] and conjugated polymers (CPs) [4]. From the early use of ruthenium(II) complexes, a significant amount of research has focussed on developing high-performance iTMC-based LEECs [11–12], with iridium(III) complexes typically showing the greatest potential. A detracting feature of many iTMC LEECs is the use of scarce noble metal complexes. Despite the enormous number of low molecular weight organic emitters designed for use in organic light-emitting diodes (OLEDs), relatively little attention has been devoted to the design of ionic small molecule (SM) [13] organic emitters for LEECs. The majority of the reported SM emitters for LEECs are fluorescent in nature and so the internal quantum efficiency (IQE) of the device is limited to 25% [13]. Thermally activated delayed fluorescent (TADF) emitters are one class of purely organic materials that can harvest triplet excitons in electroluminescent (EL) devices through a triplet to singlet reverse intersystem crossing (RISC) upconversion process [14]. Indeed, OLEDs using TADF emitters can achieve up to 100% IQE, comparable to devices using phosphorescent emitters [15].

**Figure 1:**
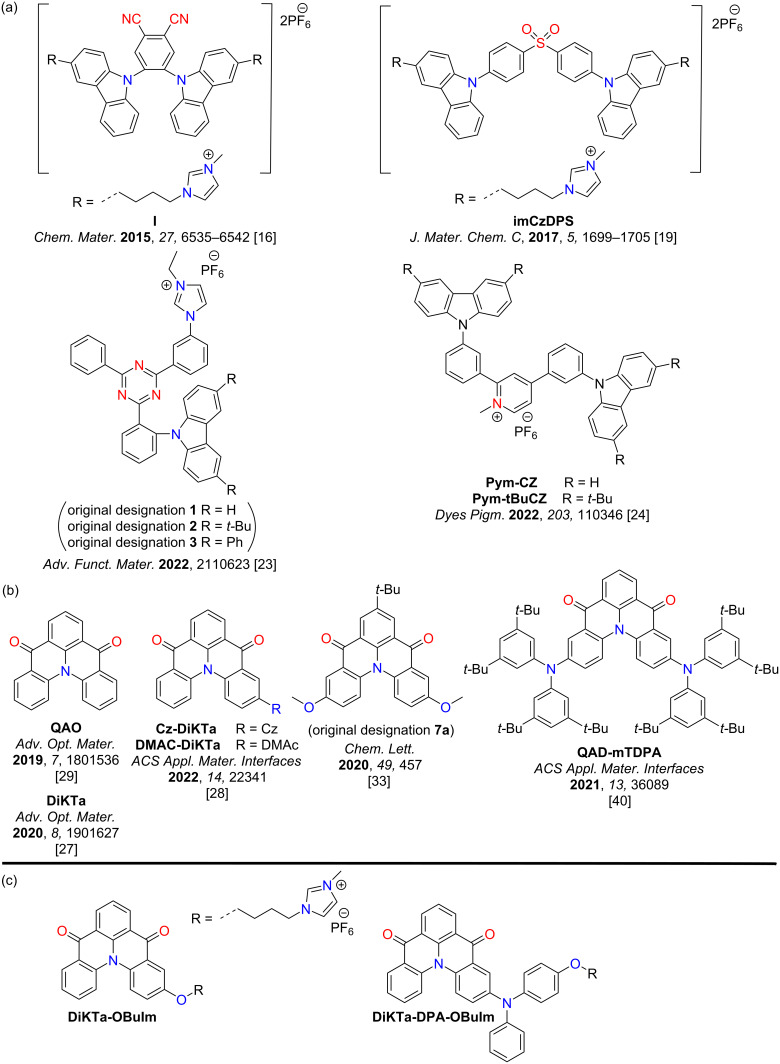
Chemical structures of (a) reported ionic TADF emitters for LEECs, (b) the MR-TADF emitter **DiKTa** and selected derivatives, and (c) the ionic emitters in this work.

Purely organic TADF emitters have not been widely investigated for use in LEECs. We reported the first organic TADF LEEC, **I** (Figure 1a, original compound **2** in [16]), in 2015 by adapting the structure of the known TADF emitter 2CzPN with imidazolium groups [16–17] (Figure 1a). The LEEC devices showed a maximum external quantum efficiency (EQE_max_) of 0.39%, a maximum brightness (*B*_max_) of 13 cd m^−2^, and a peak electroluminescence (λ_EL_) at 538 nm. The device performance suffered when the emissive layer was doped with an ionic liquid (EQE_max_ = 0.12%, *B*_max_ = 10 cd m^−2^), which was incorporated to increase charge mobility within the emissive layer. We later showed that this emitter could act as host material in combination with a cyanine dye emitter [18]. The EQE_max_ for this host–guest device was higher than for the non-doped device, at 2.0% demonstrated 100% exciton utilization efficiency in the device and efficient energy transfer from the host to the guest cyanine emitter. Deep blue emission in LEECs is challenging. We also reported a blue-emitting LEEC employing a cationic sulfone-based donor–acceptor TADF emitter, **imCzDPS** (λ_PL_ = 440 nm, Φ_PL_ = 44%, neat film) [19]. The EL of the LEEC was red-shifted at λ_EL_ of 470 nm compared to the PL. Following these initial reports Edman and co-workers demonstrated how neutral TADF small molecules [20], polymers [21], and dendrimers [22] could be employed in LEECs where the emissive layer also contained an inorganic salt and a conducting polymer. Recently, a step-change in device performance were achieved by He et al. who employed a cationic TADF compound that possesses low-lying through-space and through-bond charge transfer excited states [23]. The LEEC showed a green EL with a peak brightness of 572 cd m^−2^ and an EQE_max_ of 6.8% at 4.0 V. The half-life of their device reached 218 h at a brightness of 162 cd m^−2^. Recently, Su et al. reported two ionic TADF emitters incorporating a pyridinium moiety, **Pym-CZ** and **Pym-tBuCZ** as the acceptor and carbazole or *tert*-butylcarbazole as donor groups [24]. **Pym-CZ** showed red emission in dichloromethane (λ_PL_ = 691 nm, Φ_PL_ = 43%) and in the neat film (λ_PL_ = 583 nm, Φ_PL_ = 15%). The emission is further red-shifted and attenuated in **Pym-tBuCZ** in dichloromethane (λ_PL_ = 740 nm, Φ_PL_ = 8%) and in the neat film (λ_PL_ = 593 nm, Φ_PL_ = 6%). The LEECs with **Pym-CZ** (λ_EL_ = 599 nm, *B*_max_ = 8.69 cd m^−2^, EQE_max_ = 0.91%) and **Pym-tBuCZ** (λ_EL_ = 618 nm, *B*_max_ = 1.96 cd m^−2^, EQE_max_ = 0.05%) are the first examples of orange-red devices employing purely organic intrinsically ionic TADF emitters. Though these reports hint at the potential of TADF emitters in LEECs, the emission in these devices is typically broad, reflective of the charge transfer (CT) character of the emission, and so colour purity suffers.

Narrowband emission has, however, been demonstrated in multiresonant TADF (MR-TADF) materials. MR-TADF compounds, first introduced by Hatakeyama and co-workers, are typically p- and n-doped nanographenes [25–26]. OLEDs using MR-TADF emitters can simultaneously achieve narrowband emission and very high EQE_max_. Inspired by our recent work on neutral MR-TADF emitters for OLEDs [27–28], we designed two charged analogues of **DiKTa** [29] (Figure 1b), to make them amenable for use as emitters in LEECs, **DiKTa-OBuIm** and **DiKTa-DPA-OBuIm** (Figure 1c). In 1 wt % doped mCP films, **DiKTa-OBuIm** emits in the green region (λ_PL_ = 499 nm, Φ_PL_ = 71%, 1 wt % in mCP) and **DiKTa-DPA-OBuIm** is a red emitter (λ_PL_ = 577 nm, Φ_PL_ = 61%, 1 wt % in mCP). The presence of the DPA group in **DiKTa-DPA-OBuIm** transforms this compound from one that is MR-TADF to one that is better described as a donor–acceptor TADF, which is reflected in the red-shifted and broadened emission [28].

## Results and Discussion

**Scheme 1:**
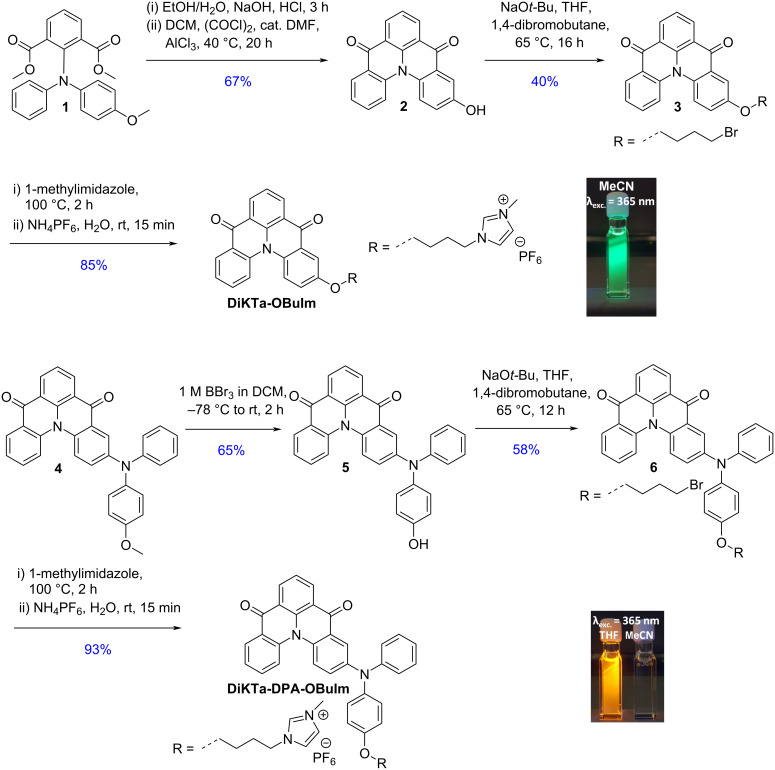
Synthesis of **DiKTa-OBuIm** and **DiKTa-DPA-OBuIm**.

**DiKTa-OBuIm** was obtained in three steps (Scheme 1) in 23% overall yield. First, hydrolysis of **1**, in situ conversion to the acyl chloride and subsequent Lewis acid-promoted Friedel–Crafts acylation reaction produced compound **2** (Scheme 1), where the AlCl_3_ was also responsible for the demethylation. Compound **2** was then subjected to monoalkylation with 1,4-dibromobutane in moderate yield, followed by a second alkylation step with 1-methylimidazole in very good yield. **DiKTa-OBuIm** was isolated as its hexafluorophosphate salt following anion metathesis with NH_4_PF_6_. **DiKTa-DPA-OBuIm** was obtained also in three steps at 35% overall yield from compound **4** using a similar synthetic strategy, which itself was synthesized from **Br-DiKTa** [28] following a Buchwald–Hartwig coupling. Details of the synthesis are found in Supporting Information File 1. The identity and purity of the molecules were verified using a combination of ^1^H and ^13^C NMR spectroscopy, high resolution mass spectrometry (HRMS) (Figures **S1–S24** in Supporting Information File 1), and melting point analysis.

**Figure 2:**
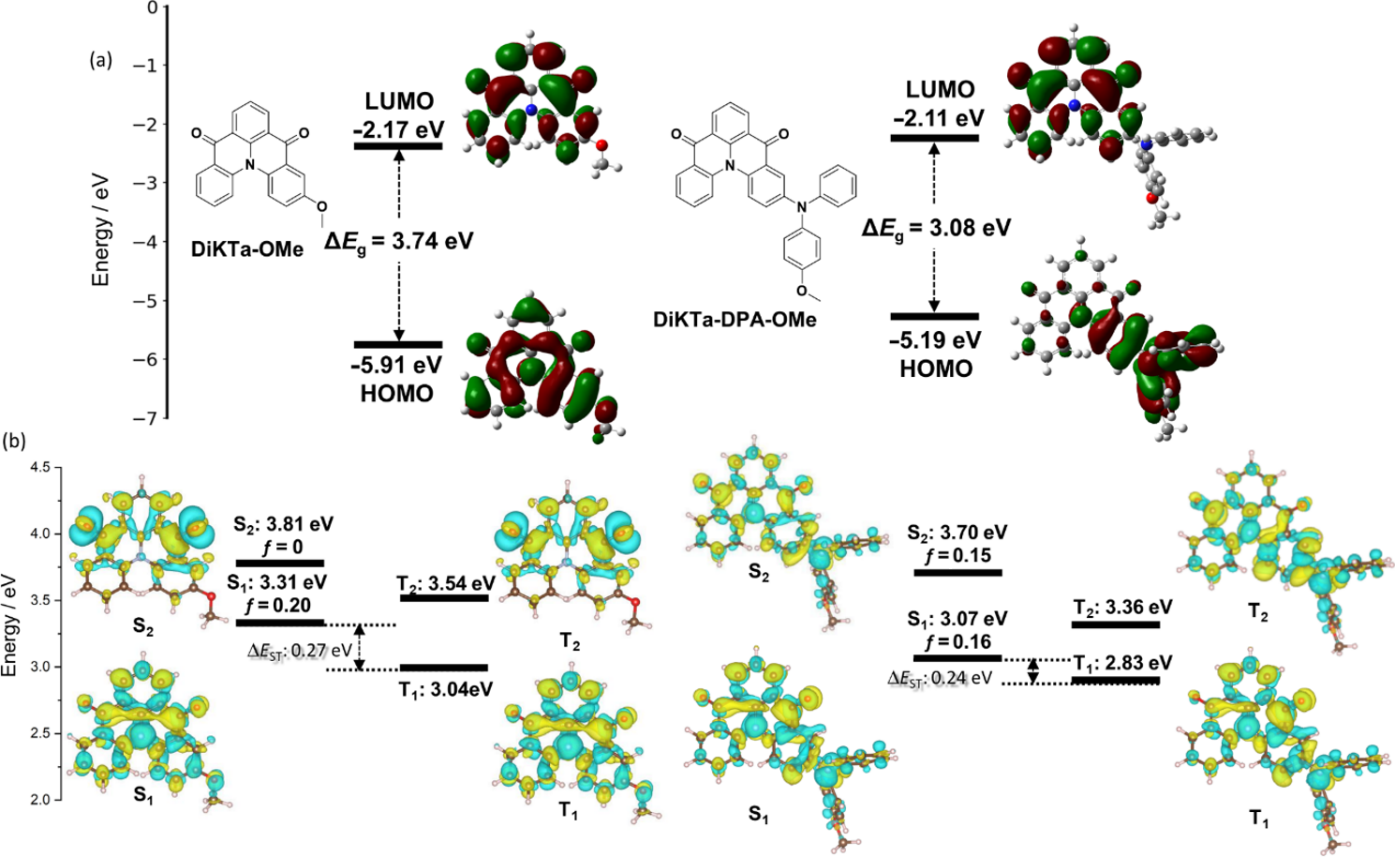
(a) HOMO and LUMO electron density distribution and orbital energies of **DiKTa-OMe** and **DiKTa-DPA-OMe** calculated at the PBE0/6-31G(d,p) level of theory in the gas phase, isovalue = 0.02; (b) difference density plots and energies for the two lowest-lying singlet and triplet excited states for **DiKTa-OMe** and **DiKTa-DPA-OMe** calculated at SCS-CC2/cc-pVDZ in the gas phase (isovalue = 0.001). The blue color represents an area of decreased electron density, and yellow represents an increased electron density between the ground and excited states; *f* denotes the oscillator strength for the transition to the excited singlet state.

We modelled the electron density distribution in **DiKTa-OBuIm** and **DiKTa-DPA-OBuIm** using density functional theory (DFT) calculations in the ground state, at the PBE0/6-31G(d,p) level of theory in the gas phase (Figure 2a). The calculations were based on model systems, **DiKTa-OMe** and **DiKTa-DPA-OMe**, respectively, wherein we replaced the imidazolium side chain of **DiKTa-OBuIm** and **DiKTa-DPA-OBuIm**, respectively, with a methyl group [30]. Compared to **DiKTa** (HOMO = −6.20 eV, LUMO = −2.23 eV, Δ*E*_g_ = 3.97 eV), both emitters possess a smaller HOMO–LUMO gap. The HOMO is more strongly affected by the incorporation of donor units [28]. For instance, in the case of **Cz-DiKTa** and **DMAC-DiKTa** the HOMO is destabilized by 0.47 eV and 0.94 eV, respectively, compared to **DiKTa** [28]. The lowest unoccupied molecular orbital (LUMO) for both compounds is localized on the **DiKTa** core (Figure S25 in Supporting Information File 1). This orbital is only slightly stabilized in **DiKTa-DPA-OMe** due to the presence of the more strongly electron-donating DPA group. The highest occupied molecular orbital (HOMO) in **DiKTa-OMe** is also localized on the **DiKTa** core and the electron density distribution of this molecule is reminiscent of that of a MR-TADF compound and is nearly identical to that of the parent emitter, **DiKTa** [27] (Figure S25 in Supporting Information File 1). There is a very large change in both the electron density distribution and the HOMO energy between the two emitters. For **DiKTa-DPA-OMe**, the HOMO is mainly localized on the DPA unit but with some delocalization onto the **DiKTa** core, resulting in a destabilization of this orbital from −5.91 eV in **DiKTa-OMe** to −5.19 eV in **DiKTa-DPA-OMe**. The HOMO–LUMO gap, Δ*E*_g_, thus decreases to 3.08 eV compared to that of **DiKTa-OMe** (3.74 eV). The excited states were modelled using spin-component scaling second-order approximate coupled-cluster (SCS-CC2) in tandem with the cc-pVDZ basis set (Table S1 in Supporting Information File 1). Figure 2b shows the difference density plots for singlet (S) and triplet (T) excited states for **DiKTa-OMe** and **DiKTa-DPA-OMe**. Compared to **DiKTa** (S_1_ = 3.45 eV, T_1_ = 3.18 eV, *f* = 0.20, Δ*E*_ST_ = 0.27 eV) [28], the lowest-lying singlet (S_1_) and triplet (T_1_) states are stabilized in the case of **DiKTa-OMe**, while the singlet–triplet energy gap, Δ*E*_ST_, remained the same at 0.27 eV. The nature of S_1_ and T_1_ resemble to those of its parent **DiKTa** and so this compound is likely to behave as a MR-TADF emitter. The nature of the S_2_ state is n–π* in **DiKTa-OMe**. The excited state picture of **DiKTa-DPA-OMe** is different to that of other reported D–A-type systems containing **DiKTa** as the acceptor [28]. Long range charge transfer is not apparent here and instead the coupled cluster calculations predict a compound that is MR-TADF but where the electron density distribution is delocalized over the entire molecule. Compared to **DiKTa-OMe**, both S_1_ and T_1_ of **DiKTa-DPA-OMe** are stabilized to 3.07 eV and 2.83 eV, respectively. The Δ*E*_ST_ decreases to 0.24 eV and there is no intermediate triplet state. The trend of stabilized S_1_ and T_1_ states when a donor group decorates the **DiKTa** core (S_1_ = 3.45 eV, T_1_ = 3.18 eV) has been previously observed in reported emitters such as **Cz-DiKTa** (S_1_ = 3.35 eV, T_1_ = 3.09 eV) and **DMAC-DiKTa** (S_1_ = 3.43 eV, T_1_ = 3.17 eV) [28]. We also calculated the charge transfer character of each excited state, focussing on the distance of charge transfer (D_CT_). When considering the S_1_ excited state, there is an increase in CT character moving from **DiKTa**, **DiKTa-OMe**, and **DiKTa-DPA-OMe** (D_CT_ = 1.45 Å, 1.81 Å, and 3.34 Å, respectively) reflected in the increased donor strength.

**Figure 3:**
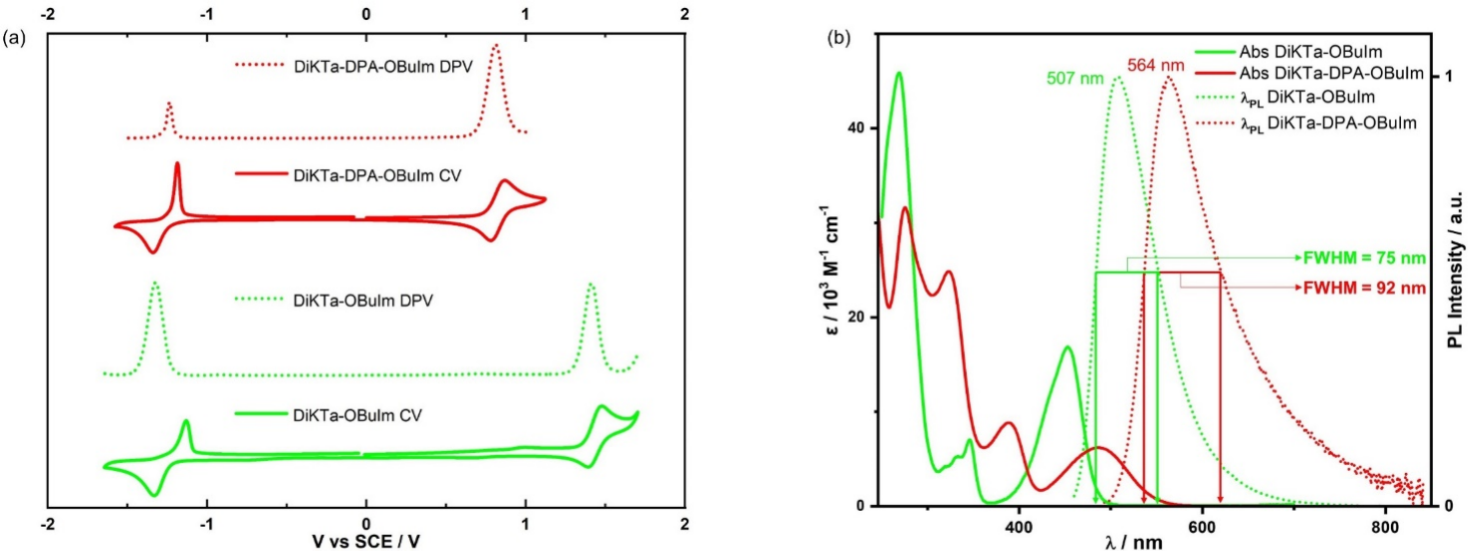
(a) Cyclic and differential pulse voltammograms measured in degassed MeCN with 0.1 M [*n-*Bu_4_N]PF_6_ as the supporting electrolyte and Fc^+^/Fc as the internal reference (0.38 V vs SCE) [32]. Scan rate = 100 mV s^−1^; (b) solution-state photophysical measurements: absorption and steady-state emission spectra at 300 K measured in MeCN. λ_exc_ = 453 nm for **DiKTa-OBuIm** and λ_exc_ = 488 nm for **DiKTa-DPA-OBuIm**.

The electrochemical properties of **DiKTa-OBuIm** and **DiKTa-DPA-OBuIm** were investigated by cyclic voltammetry (CV) and differential pulse voltammetry (DPV) in acetonitrile with 0.1 M tetra-*n*-butylammonium hexafluorophosphate as the supporting electrolyte (Figure 3a and Table S2 in Supporting Information File 1). The oxidation and reduction of both emitters showed good reversibility, which is beneficial for better performance in LEEC devices [31]. The oxidation potentials, *E*_ox_, determined from the peak value of the first DPV curve are 1.05 V and 0.44 V for **DiKTa-OBuIm** and **DiKTa-DPA-OBuIm**, respectively, which correspond to HOMO energy levels of −5.85 eV and −5.24 eV, respectively. The trend of a destabilized HOMO energy level from **DiKTa-OBuIm** to **DiKTa-DPA-OBuIm** is predicted by DFT calculations. **DiKTa** possesses an oxidation potential of 1.66 V and an associated HOMO energy level of −5.93 eV. The reduction potentials, *E*_red_, are −1.67 V and −1.61 V, respectively, for **DiKTa-OBuIm** and **DiKTa-DPA-OBuIm**. The corresponding LUMO levels are −3.13 eV and −3.18 eV for **DiKTa-OBuIm** and **DiKTa-DPA-OBuIm**, respectively. The LUMO values of both emitters match that of **DiKTa** (−3.11 eV), which suggests that reduction occurs on the **DiKTa** core in both compounds, a contention corroborated by the DFT calculations. The electrochemical gap reduced from 2.72 V in **DiKTa-OBuIm** to 2.06 V in **DiKTa-DPA-OBuIm**, a trend that is in line with the DFT calculations.

Figure 3b shows the solution-state photophysical properties of **DiKTa-OBuIm** and **DiKTa-DPA-OBuIm** in acetonitrile and the data are compiled in Table 1. The lowest energy absorption band for **DiKTa-OBuIm** at 453 nm (ε = 17 × 10^3^ M^−1^ cm^−1^) is red-shifted and slightly more intense than that of the parent **DiKTa** at 436 nm, (ε = 14 × 10^3^ M^−1^ cm^−1^) [27] owing to the increased conjugation in **DiKTa-OBuIm**. For the emitter **7a** (Figure 1b) [33] reported by Yan et al. the red-shift of the lowest energy absorption band was more pronounced than that in **DiKTa-OBuIm**. This band is assigned to a short-range charge transfer transition (SRCT) that is a hallmark characteristic in MR-TADF compounds [28]. The Stokes shift is 54 nm (2361 cm^−1^) for **DiKTa-OBuIm**. The lowest energy absorption band in **DiKTa-DPA-OBuIm** is red-shifted and less intense (ε = 6 × 10^3^ M^−1^ cm^−1^) compared to **DiKTa-OBuIm**, in line with its decreased oscillator strength (vide supra). According to the calculations (vide supra), the S_1_ excited state is also SRCT, but with larger long-range charge transfer (LRCT) content. Owing to the relative flexibility around the DPA donor unit, the Stokes shift is larger at 75 nm (2761 cm^−1^). **DiKTa-OBuIm** and **DiKTa-DPA-OBuIm** exhibited broad green (λ_PL_ = 507 nm, FWHM = 75 nm) and red (λ_PL_ = 563 nm, FWHM = 92 nm) emissions in MeCN, respectively, which is larger than **DiKTa** (46 nm in MeCN) [27] in line with the greater LRCT character for these emitters; this observation has been noted for other donor decorated MR-TADF emitters [34–36]. The photoluminescence quantum yield, Φ_PL_, in MeCN for **DiKTa-OBuIm** is 48% which decreases in air to 34%. The emission is much weaker in **DiKTa-DPA-OBuIm**, reflecting both the smaller oscillator strength of the transition to S_1_ and the greater non-radiative decay due to the energy gap law (Φ_PL_ = 11% and 7% under vacuum and in air, respectively) in MeCN [37]. The S_1_ and T_1_ levels were measured from the onsets of fluorescence (2.66 eV) and phosphorescence spectra (2.41 eV) in 2-MeTHF glass at 77 K (Figure S26, Supporting Information File 1). **DiKTa-OBuIm** possesses a Δ*E*_ST_ of 0.25 eV. Unfortunately, **DiKTa-DPA-OBuIm** was insoluble in 2-MeTHF and so the measurement could not be made. No delayed component was observed in MeCN solution under vacuum for either of the compounds (Figure S27 in Supporting Information File 1).

**Table 1:** Photophysical properties of **DiKTa-OBuIm** and **DiKTa-DPA-OBuIm**.

Compound	Medium	λ_Abs_^a^ [nm]	λ_PL_^b^ [nm]	FWHM^c^ [nm]	*E*_S1_^d^ [eV]	*E*_T1_^d^ [eV]	*∆E*_ST_^e^ [eV]

**DiKTa-OBuIm**	sol.^f^	453 (17)	507	75	2.66	2.41	0.25
	film^g^	–	500	66	2.65	2.45	0.20
**DiKTa-DPA-OBuIm**	sol.^f^	488 (6)	563	92	–	–	–
	film^g^	–	578	95	2.40	2.21	0.19

	Φ_PL_^h^ [%]	τ_p_^i^ [ns]	τ_d_^i^ [μs]	*k*_ISC_^j^ [s^−1^] (×10^7^)	*k*_RISC_^j^ [s^−1^] (×10^3^)	*k*_s_r_^j^ [s^−1^] (×10^7^)	*k*_s_nr_^j^ [s^−1^] (×10^7^)

**DiKTa-OBuIm**	48^a^	14.3^a^	–	–	–	–	–
	71 (57)^b^	8.7 ^b^	316.6^b^	3.59 ± 1.3	2.85 ± 1.1	6.60	2.69
**DiKTa-DPA-OBuIm**	11^a^	12.7^a^	–	–	–	–	–
	61 (53)^b^	14.1^b^	241.7^b^	2.21 ± 1.2	3.04 ± 1.7	3.78	2.38

^a^Lowest energy absorbance band, absorptivity (ε) in parentheses (/ × 10^3^ M^−1^ s^−1^). ^b^Steady-state emission maximum at 300 K; λ_exc_ = 340 nm. ^c^Full width at half maximum of the emission peak. ^d^S_1_ and T_1_ energies were obtained from the onsets of the respective prompt fluorescence (delay: 1 ns; gate time: 100 ns) and phosphorescence spectra (delay: 1 ms; gate time: 9 ms) at 77 K; λ_exc_ = 343 nm. ^e^Δ*E*_ST_ = *E*(S_1_) − *E*(T_1_). ^f^In MeCN solutions (10^−6^ M). ^g^Measured in spin-coated thin films consisting of 1.0 wt % emitter in mCP; λ_exc_ = 340 nm. ^h^Φ_PL_ in solutions were measured by the relative method using quinine sulfate as a standard (Φ_r_ = 54.6% in 1 N H_2_SO_4_) [38], while absolute Φ_PL_ of thin films were measured using an integrating sphere; λ_exc_ = 340 nm under nitrogen and the values in parentheses are in the presence of O_2_. ^i^Prompt and delayed lifetimes in solutions and thin films obtained by TCSPC and MCS, λ_exc_ = 379 nm. ^j^Intersystem and reverse intersystem crossing rates were calculated using the steady-state approximation method as described in literature [39].

**Figure 4:**
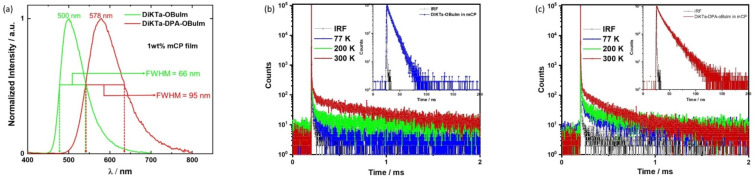
(a) Steady-state emission spectra of **DiKTa-OBuIm** and **DiKTa-DPA-OBuIm** in 1 wt % doped mCP films, λ_exc._ = 340 nm; (b) temperature-dependent time resolved PL decays of **DiKTa-OBuIm** in 1 wt % doped mCP films. Inset: prompt PL decay of **DiKTa-OBuIm;** (c) temperature-dependent time resolved PL decays of **DiKTa-DPA-OBuIm** in 1 wt % doped mCP films. Inset: prompt PL decay of **DiKTa-DPA-OBuIm**, λ_exc._ = 379 nm.

The thin film PL behavior of both emitters was then assessed in 1 wt % doped film in 1,3-di-9-carbazolylbenzene (mCP) (Figure 4). At this doping concentration, the photophysical properties should reflect monomolecular entities. Emission was observed at 500 nm (FWHM = 66 nm) and 578 nm (FWHM = 95 nm) for **DiKTa-OBuIm** and **DiKTa-DPA-OBuIm**, respectively. The emission spectrum of **DiKTa-OBuIm** is slightly blue-shifted and narrower than that in MeCN, which is expected due to the higher polarity of the solvent than mCP. Surprisingly, for **DiKTa-DPA-OBuIm** the emission is red-shifted by 14 nm, and with negligible change in the FWHM. This suggests that the conformation of the emitter in the solid state is slightly more conjugated than that in solution or that there are specific host–guest interactions with the DPA unit that perturbs the energy of the excited state. The emission is broader than that of a structurally similar emitter, **QAD-mTDPA**, a derivative of **DiKTa** containing two DPA substituents, reported by Zhang et al. [40] The structure of **QAD-mTDPA** (λ_PL_ = 587 nm, FWHM = 62 nm, Φ_PL_ = 97%, Δ*E*_ST_ = 0.33 eV, τ_D_ = 269 μs, 1.5 wt % CBP) is shown in Figure 1b. Both emitters showed red-shifted and broadened emission compared to that of **DiKTa** (λ_PL_ = 466 nm, FWHM = 40 nm, Φ_PL_ = 70%, Δ*E*_ST_ = 0.20 eV, τ_D_ = 168 μs, 2 wt % mCP) in the same host [28]. Both emitters exhibited high Φ_PL_ values in the mCP film at 71% and 61% under nitrogen, and these reduced to 57% and 53% in air for **DiKTa-OBuIm** and **DiKTa-DPA-OBuIm**, respectively. As neat thin films, the emission for both compounds are red-shifted and significantly quenched (Figure S28 in Supporting Information File 1); indeed, the Φ_PL_ for the neat film of **DiKTa-OBuIm** is only 9% while we could not ascertain a reliable value for **DiKTa-DPA-OBuIm**. Severe aggregation-caused quenching of the emission in the neat film was also observed for **DiKTa** (Φ_PL_ = 11%, under N_2_) [27]. The S_1_ and T_1_ levels were measured from the onsets of fluorescence and phosphorescence spectra in the 1 wt % doped mCP film at 77 K (Figure S29 in Supporting Information File 1). The corresponding Δ*E*_ST_ values are 0.20 eV and 0.19 eV, respectively, for **DiKTa-OBuIm** and **DiKTa-DPA-OBuIm**, which are nearly same to that reported for **DiKTa** (Δ*E*_ST_ = 0.20 eV) [28]. Experimental Δ*E*_ST_ values are smaller than those computationally predicted (0.27 eV and 0.24 eV, respectively for **DiKTa-OBuIm** and **DiKTa-DPA-OBuIm**). However, the trend of decreasing Δ*E*_ST_ is in line to the findings from DFT. The temperature dependent time-resolved PL decays in the 1 wt % doped mCP films are presented in Figure 4b and c. Both emitters show prompt and delayed emission components with an enhancement of the delayed emission with increasing temperature, a feature of TADF. Unlike the delayed emission lifetime of **DiKTa** (15 μs in 3.5 wt % mCP, 23 μs in PhMe) [27], and its derivatives such as **Cz-DiKTa** (τ_D_ = 196 μs, 2 wt % mCP), **DMAC-DiKTa** (τ_D_ = 6.6 μs, 2 wt % mCP), and **QAD-mTDPA** (τ_D_ = 168 μs, in 2 wt % mCP) in Figure 1b [28,40], the delayed lifetimes from **DiKTa-OBuIm** and **DiKTa-DPA-OBuIm** are long at 317 μs and 242 μs, respectively. RISC rate constants, *k*_RISC_, were calculated for both emitters, which are 2.85 × 10^3^ s^−1^ and 3.04 × 10^3^ s^−1^, respectively for **DiKTa-OBuIm** and **DiKTa-DPA-OBuIm**, compared to that of **DiKTa** (4.6 × 10^4^ s^−1^) in toluene [27,39].

### Light-emitting electrochemical cells

**Figure 5:**
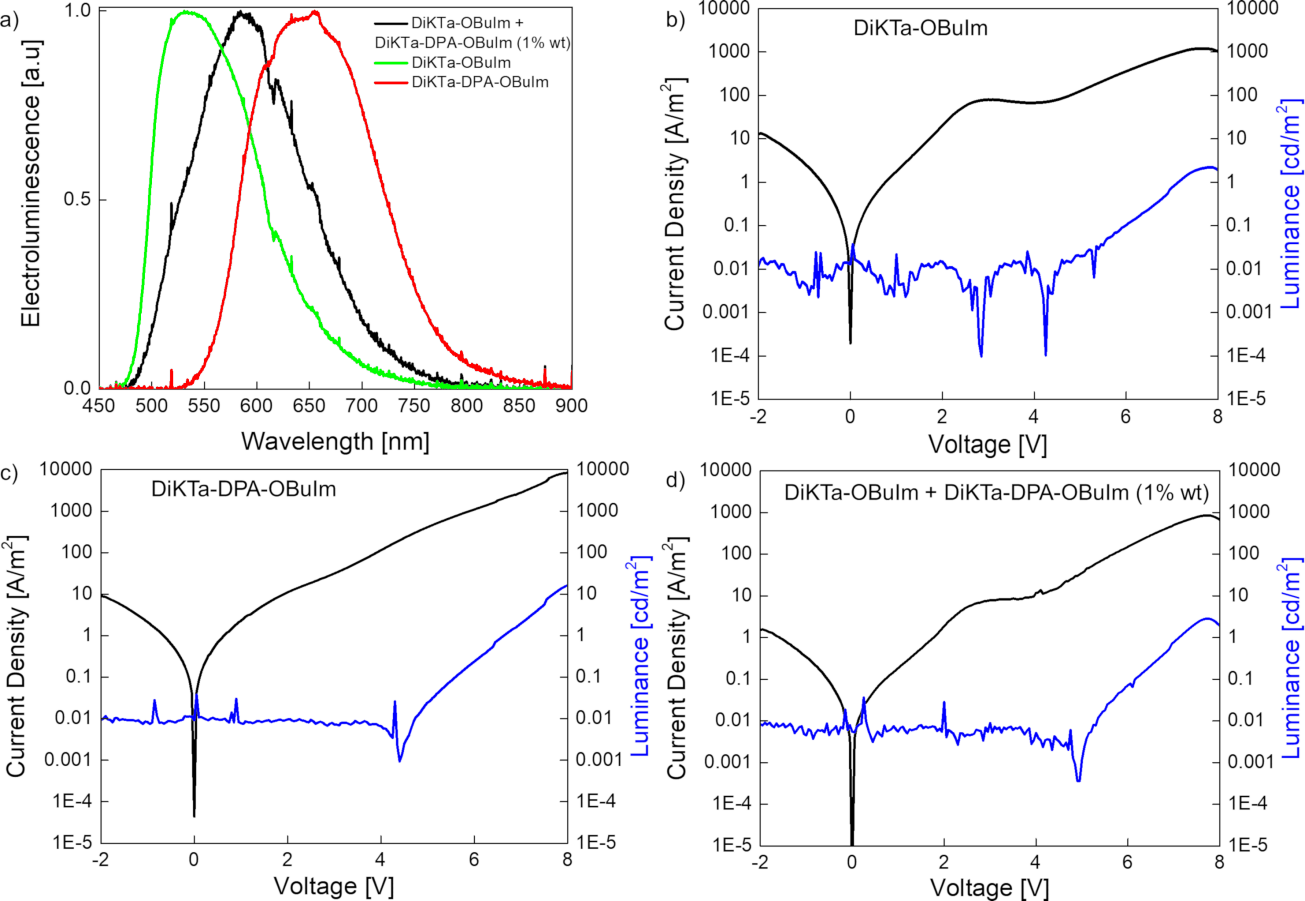
(a) Electroluminescence spectra of **DiKTa-OBuIm** (green curve), **DiKTa-DPA-OBuIm** (red curve) and 1% of **DiKTa-DPA-OBuIm** in **DiKTa-OBuIm** (black curve). Current (black) and luminance (blue) versus voltage (*JVL*) sweep (from −2 to 8 V) of (b) **DiKTa-OBuIm**, (c) **DiKTa-DPA-OBuIm**, and (d) 1% of **DiKTa-DPA-OBuIm** in **DiKTa-OBuIm**.

LEECs were fabricated using **DiKTa-OBuIm** and **DiKTa-DPA-OBuIm** as emitters. The device stack was the following: ITO/PEDOT:PSS/emitter/Al (where ITO is indium tin oxide; PEDOT:PSS is poly(3,4-ethylenedioxythiophene):poly(styrenesulfonate)). The PEDOT:PSS and the emitter layers were prepared from solution and the device was finished with an evaporated Al top contact. Details of the LEEC fabrication can be found in the General Methods section of Supporting Information File 1. Driven by their promising Φ_PL_ LEEC devices using **DiKTa-OBuIm** and **DiKTa-DPA-OBuIm** as 1 wt % doped films in mCP as the emitter layer were prepared. The devices showed no turn-on, both in lifetime measurements and in current density and luminance versus voltage sweeps (*JVL*) up to 8 V. Most likely the low content of ionic species in the neutral matrix hindered the required ionic transport for LEEC operation. To solve this, we fabricated devices adding an ionic liquid (lithium hexafluorophosphate (LiPF_6_) or 1-butyl-3-methylimidazolium hexafluorophosphate (BMIM:PF_6_) in a 4 to 1 molar ratio) and, in some cases, an electrolyte matrix (PEO (polyethylene oxide)), to improve the ionic mobility on the active film [5,41]. However, despite these efforts, still no emission was observed when the devices were biased. Next, neat films of **DiKTa-OBuIm** and **DiKTa-DPA-OBuIm** were directly used as active layers. Non-doped small molecule films have shown recently promising results in LEEC devices [42]. As both emitters are ionic, in principle there is no need to incorporate additional mobile ions. A host–guest approach, using 1 wt % of **DiKTa-DPA-OBuIm** in **DiKTa-OBuIm** was also used, the latter acting as a host matrix for the former. The electroluminescence (EL) of the three device stacks is shown in Figure 5a. Similar to the PL, the EL spectra are broad and unstructured. The EL of **DiKTa-OBuIm** and **DiKTa-DPA-OBuIm** occurs at λ_EL_ of 534 and 656 nm, respectively. Both neat-film EL spectra are red-shifted from the solution state and the 1 wt % in mCP film PL spectra. The origin of this red shift could be ascribed to the presence of emissive aggregates in the emissive layer [19]. Interestingly, in the host–guest system the energy transfer is not complete and both molecules are responsible for the electroluminescence, with a λ_EL_ at 586 nm, between the emission of the neat films. *JVL* characterization (from −2 to 8 V) was carried out on the three stacks (Figure 5b–d). As it can be seen, the current density reaches high values, and the injection is primarily dominated by ohmic behavior. The device with **DiKTa-DPA-OBuIm** shows a steeper injection reaching values of 10,000 A m^−2^ at 8 V when compared with the device with **DiKTa-OBuIm**, which shows a current density of 1000 A m^−2^ at the same voltage value. The current density in the device with the host–guest system is dominated by the presence of **DiKTa-OBuIm**. Light emission is detected at around ≈5 V, with values of 15 cd m^−2^ for the device with **DiKTa-DPA-OBuIm** and around 2 cd m^−2^ for the devices with **DiKTa-OBuIm** and the host–guest system, each at 8 V. From the EL spectra it is possible to estimate the external quantum efficiency (EQE) values; however, they are also highly affected by the luminance levels, giving as a result very low efficiencies (<0.01%).

## Conclusions

Two new ionic TADF emitters were designed and synthesized for LEECs application using a known MR-TADF emitter **DiKTa**. Our MR-TADF green emitter, **DiKTa-OBuIm** exhibited efficient green luminescence and TADF in 1 wt % mCP film (λ_PL_ = 499 nm, FWHM = 66 nm, Φ_PL_ = 71%, τ_d_ = 317 μs, *k*_RISC_ = 2.85 × 10^3^ s^−1^). This emitter represents a rare example of an ionic MR-TADF emitter for LEEC applications. The red emitter, **DiKTa-DPA-OBuIm**, was obtained by coupling a methoxy-modified diphenylamine unit onto the **DiKTa** fragment. Addition of a donor unit red-shifted the emission to red region with TADF (λ_PL_ = 577 nm, FWHM = 95 nm, Φ_PL_ = 61%, τ_d_ = 242 μs, *k*_RISC_ = 3.04 × 10^3^ s^−1^, 1 wt % in mCP). Different strategies were explored to prepare LEECs based on **DiKTa-OBuIm** and **DiKTa-DPA-OBuIm** as emitters. The devices showed green and red emission, respectively.

## Supporting Information

The research data supporting this publication can be accessed at https://doi.org/10.17630/6ef45b8f-579d-4075-891e-595516c56e47.

File 1 ^1^H NMR and ^13^C NMR spectra, GC–MS, and HRMS; supplementary computational data and coordinates; additional photophysical.
