# Supplementary material for: Ionic multiresonant thermally activated delayed fluorescence emitters for light emitting electrochemical cells
Source: Beilstein J Org Chem. 2022 Sep 22;18:1311–21. doi: 10.3762/bjoc.18.136 (PMC9520854; doi:10.3762/bjoc.18.136)
Supplement: File 1 — 1H NMR and 13C NMR spectra, GC–MS, and HRMS; supplementary computational data and coordinates; additional photophysical. [file Beilstein_J_Org_Chem-18-1311-s001.pdf]

BEILSTEIN JOURNAL OF ORGANIC CHEMISTRY

Supporting Information

for

Ionic multiresonant thermally activated delayed fluorescence emitters for light emitting electrochemical cells

Merve Karaman, Abhishek Kumar Gupta, Subeesh Madayanad Suresh, Tomas Matulaitis, Lorenzo Mardegan, Daniel Tordera, Henk J. Bolink, Sen Wu, Stuart Warriner, Ifor D. Samuel and Eli Zysman-Colman

Beilstein J. Org. Chem. **2022,** *18,* 1311–1321. doi:10.3762/bjoc.18.136

^{1}H NMR and ^{13}C NMR spectra, GC–MS, and HRMS; supplementary computational data and coordinates; additional photophysical

License and Terms: This is a supporting information file under the terms of the Creative Commons Attribution License (https://creativecommons.org/licenses/by/4.0). Please note that the reuse, redistribution and reproduction in particular requires that the author(s) and source are credited and that individual graphics may be subject to special legal provisions.
The license is subject to the *Beilstein Journal of Organic Chemistry* terms and conditions: (https://www.beilstein-journals.org/bjoc/terms)

Table of contents

	Page
**General methods**	**S1**
**Experimental section**	**S6**
**^{1}H, ^{13}C NMR spectra and HRMS**	**S13**
**Supplementary calculations**	**S25**
**Photophysical characterization**	**S26**
**References**	**S27**

General methods

General Synthetic Procedures. The following starting materials were synthesised according to literature procedures, 2-bromoisophthalic acid[1], dimethyl 2-bromoisophthalic acid [1], 4-methoxy-*N*-phenyl-aniline [2], and **Br-DiKTa** [3]. All other reagents and solvents were obtained from commercial sources and used as received. Air-sensitive reactions were performed under a nitrogen atmosphere using Schlenk techniques, no special precautions were taken to exclude air or moisture during work-up and crystallisation. Flash column chromatography was carried out using silica gel (Silica-P from Silicycle, 60 Å, 40–63 µm). Analytical thin-layer-chromatography (TLC) was performed with silica plates with aluminum backings (250 µm with F-254 indicator). TLC visualisation was accomplished by a 254/365 nm UV lamp. ^{1}H NMR spectra were recorded on a Bruker Advance spectrometer (500 or 700 MHz for ^{1}H and 125 or 175 for ^{13}C). The following abbreviations have been used for multiplicity assignments: "s" for singlet, "d" for doublet, and "dd" for doublet of doublet. ^{1}H NMR spectra were referenced to residual solvent peaks with respect to TMS ($\delta = 0$ ppm). Melting points were measured using open-ended capillaries on an Electrothermal 1101D Mel-Temp apparatus and are uncorrected. High-resolution mass spectrometry (HRMS) was performed by School of chemistry at the University of Leeds.

Photophysical measurements. Optically dilute solutions of concentrations on the order of 10^{-5} to 10^{-6} M were prepared in spectroscopic or HPLC grade solvents for absorption and emission analyses. Absorption spectra were recorded at room temperature on a Shimadzu UV-2600 double beam spectrophotometer with a 1 cm quartz cuvette. Molar absorptivity determination was verified by linear regression analysis of values obtained from at least five independent solutions at varying concentrations ranging from 10^{-5} M to 10^{-6} M. Steady-state emission and excitation spectra and time-resolved emission decays were recorded at 298 K using an Edinburgh Instruments FS5 spectrofluorometer. Samples were excited at 378 nm for steady-state measurements and at 378 nm for time-resolved measurements.

For photoluminescence quantum yield measurements, degassed solutions were prepared via three freeze-pump-thaw cycles and spectra were taken using home-made Schlenk quartz cuvette. Photoluminescence quantum yields for solutions were determined using the optically dilute method [4] in which four sample solutions with absorbances of ca. 0.19, 0.15, 0.09, and 0.05 for **DiKTa-OBuIm** and ca. 0.21, 0.17, 0.10, and 0.05 for **DiKTa-DPA-OBuIm** were used. The Beer–Lambert law was found to remain linear at the concentrations of the solutions. For each sample, linearity between absorption and emission intensity was verified through linear regression analysis with the Pearson regression factor (R^2) for the linear fit of the data set surpassing 0.9. Individual relative quantum yield values were calculated for each solution and the values reported represent the slope obtained from the linear fit of these results. The quantum yield of the sample, Φ_{PL}, can be determined by the equation $\Phi_{PL} = (\Phi_r * \frac{A_r}{A_s} * \frac{I_s}{I_r} * \frac{n_s^2}{n_r^2})^2$, where *A* stands for the absorbance at the excitation wavelength (λ_{exc}: 379 nm), *I* is the integrated area under the corrected emission curve and *n* is the refractive index of the solvent with the subscripts "s" and "r" representing sample and reference respectively. Φ_r is the absolute quantum yield of the external reference quinine sulphate (Φ_r = 54.6% in 1 N H_2SO_4) [5], The experimental uncertainty in the emission quantum yields is conservatively estimated to be 10%, though we have found that statistically we can reproduce Φ_{PL} values to 3% relative error.

Spin-coated thin films were used to measure thin film photophysical properties in the solid state. An integrating sphere (Hamamatsu, C9920-02) was employed for photoluminescence quantum yield measurements for thin film samples. Time-resolved PL measurements of the thin films (film of emitters doped in mCP coated on sapphire substrate and annealed at 140 °C for 3 min) were carried out using the time-correlated single photon counting system (TCSPC) and multichannel scaling (MCS). The film samples were excited at 379 nm by a pulsed laser diode (Picoquant, model PLS 370) and were kept in a vacuum of $< 8 \times 10^{-4}$ mbar.

Prompt fluorescence and phosphorescence spectra were obtained from dilute samples in 2-methyltetrahydrofuran and spin-coated films at 77 K and the singlet–triplet splitting energy values, ΔE_{ST}, were estimated from the difference in the onsets of the corresponding spectra. For ΔE_{ST} measurements of film samples were loaded inside a cold finger cryostat (Oxford Instruments) and placed under vacuum, with temperature control from 300 K – 77 K, while an open Dewar was used for solution samples. All samples were photoexcited using the third harmonic emission (343 nm) from a femtosecond Nd:YAG laser, which originally emits at 1030 nm (Orpheus-N, model: PN13F1). Emission from the samples was focused onto a spectrograph (Chromex imaging, 250is spectrograph) and detected on a sensitive gated iCCD camera (Stanford Computer Optics, 4Picos) having sub-nanosecond resolution. Phosphorescence spectra were measured 1 ms after the excitation of the Nd:YAG laser with iCCD exposure time of 8.5 ms. Prompt fluorescence spectra were measured 1 ns after the excitation of the femtosecond laser with iCCD exposure time of 100 ns.

Fitting of the time-resolved luminescence measurements and relative rates: Time-resolved PL measurements were fitted to a sum of exponentials decay model, with chi-squared (χ^2) values between 1 and 2, using the EI software. Each component of the decay is assigned a weight, (w_i), which is the contribution of the emission from each component to the total emission.

The average lifetime was then calculated using the following:

- Two exponential decay model:

$$\tau_{AVG} = \tau_1 w_1 + \tau_2 w_2$$

with weights defined as $w_1 = \frac{A1\tau_1}{A1\tau_1 + A2\tau_2}$ and $w_2 = \frac{A2\tau_2}{A1\tau_1 + A2\tau_2}$ where A1 and A2 are the preexponential-factors of each component.

- Three exponential decay model:

$$\tau_{AVG} = \tau_1 w_1 + \tau_2 w_2 + \tau_3 w_3$$

with weights defined as $w_1 = \frac{A1\tau_1}{A1\tau_1 + A2\tau_2 + A3\tau_3}$, $w_2 = \frac{A2\tau_2}{A1\tau_1 + A2\tau_2 + A3\tau_3}$ and $w_3 = \frac{A3\tau_3}{A1\tau_1 + A2\tau_2 + A3\tau_3}$ where A1, A2 and A3 are the preexponential-factors of each component.

- Relative rates k_{risc}, k_{isc}, $k^{s}{}_{,r}$ and $k^{s}{}_{nr}$ were calculated according to reported literature [6].

Electrochemistry measurements. Cyclic Voltammetry (CV) analysis was performed on an Electrochemical Analyzer potentiostat model 620E from CH Instruments at a sweep rate of 100 mV/s. Differential pulse voltammetry (DPV) was conducted with an increment potential of 0.004 V and a pulse amplitude, width, and period of 50 mV, 0.05, and 0.5 s, respectively. Samples were prepared as acetonitrile (MeCN) solutions, which were degassed by sparging with MeCN-saturated nitrogen gas for 5 minutes prior to measurements. All measurements were performed using 0.1 M MeCN solution of tetra-*n*-butylammonium hexafluorophosphate ([nBu$_4$N]PF$_6$]). An Ag/Ag$^+$ electrode was used as the reference electrode while a platinum electrode and a platinum wire were used as the working electrode and counter electrode, respectively. The HOMO and LUMO energies were determined using the relation $E_{HOMO/LUMO} = -(E_{ox} / E_{red} + 4.8)$ eV [7], where E_{ox} and E_{red} are the anodic and cathodic peak potentials, respectively calculated from DPV related to Fc/Fc$^+$. $E_{H-L} = |E_{HOMO} - E_{LUMO}|$.

*Quantum chemical calculations***.** The calculations were performed with the Gaussian 16 revision A03 suite for the density functional theory (DFT) [8] and with the Turbomole/7.4 package for SCS-CC2 calculations [9]. We optimised the ground state using PBE0 [10] with the 6-31G(d,p) [11] basis set. Vertical excited states were performed based on the ground state optimised structure using spin-component scaling coupled-cluster singles-and-doubles model (SCS-CC2) with the cc-pVDZ basis set [12], computing the two first singlet (S_1 and S_2) [13] and two first triplet excited states (T_1 and T_2) [14]. Molecular orbitals were visualised using GaussView 6.0 [15]. Difference density plots were used to visualise change in electronic density between the ground and excited state and were obtained using the

VESTA package [16]. Charge transfer descriptors, distance of charge transfer (D_{CT}) was calculated from the difference density plots using Multiwfn software package [17].

Device fabrication and characterization. All devices were fabricated by a hybrid of solution-processing and thermal evaporation of the top Al electrode. The final architecture of the devices was ITO/PEDOT:PSS/Active Layer/Al. Solutions of the emitters **DiKTa-OBuIm** and **DiKTa-DPA-OBuIm** were made at a concentration of 20 mg/mL in acetonitrile (MeCN). When used, solutions of mCP (1,3-bis(*N*-carbazolyl)benzene), PEO (poly(ethylene) oxide), [BMIM]PF_6 (1-butyl-3-methylimidazolium hexafluorophosphate), and $LiPF_6$ (lithium hexafluorophosphate) were made at a concentration of 10 mg/mL in either MeCN or dichloromethane (DCM). In mCP as host, the emitters were mixed at a concentration of 1 wt % while [BMIM]PF_6 was added at a molar ratio of 1:0.25 with respect to the emitter. PEO was used at three different mass ratios: 1:0.5, 1:1, and 1:1.25, while $LiPF_6$ was kept constant at 1:0.25 molar ratio. Pre-patterned indium tin oxide (ITO)-coated glass plates were used as transparent conductive substrates. They were subsequently cleaned ultrasonically in water-soap, water, and 2-propanol baths. After drying, the substrates were placed in a UV-ozone cleaner (Jelight 42-220) for 20 min. The ITO substrates were first coated with PEDOT:PSS at 4000 rpm and then with the emitter solutions at 2000 rpm. Thicknesses of 80 nm and 80–100 nm were obtained, respectively. The thin films were annealed at 90 °C for 30 min under an inert atmosphere. Then an Al cathode was deposited via thermal evaporation using a shadow mask. The final active area of the cells was 6 mm^2. The electroluminescence spectra were measured with an optical fiber connected to an Avantes AvaSpec-2048L spectrometer while driving the cell with a Botest OLT OLED Lifetime-Test system. The current density and luminance versus voltage (*JVL*) sweeps data was measured by using a Keithley and a picoamperometer. The lifetime characterisation was carried out by applying a pulsed current density of 50 A m^{-2} while monitoring the voltage and luminance versus time by using a True Color Sensor MAZeT (MTCSiCT sensor) with a Botest OLT OLED Lifetime-Test system. The applied pulsed current consisted in block waves at a frequency of 1000 Hz with a duty cycle of 50%. As a result, the average current density and voltage were obtained by multiplying the values by the time-on (0.5 s) and dividing by the total cycle time (1 s). The characterisation was conducted under nitrogen atmosphere.

Synthesis

Dimethyl 2-((4-methoxyphenyl)(phenyl)amino)isophthalate (1)

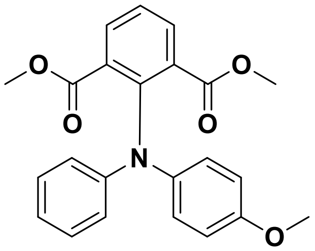

4-Methoxy-*N*-phenylaniline (2.00 g, 10.0 mmol, 1 equiv), dimethyl 2-bromoisophthalate (3.00 g, 11.0 mmol, 1.1 equiv), K_2CO_3 (6.94 g, 50.2 mmol, 5.0 equiv), CuI (191 mg, 1.00 mmol, 0.10 equiv), CuSn (183 mg, 1.00 mmol, 0.10 equiv), 2,2,6,6-tetramethylheptane-3,5-dione (0.21 mL, 1.0 mmol, 0.10 equiv), and di-*n*-butyl ether (10 mL) were mixed in a Schlenk tube under nitrogen. The reaction mixture was stirred at 150 °C for 3 h. The mixture was cooled to rt and diluted with DCM (100 mL) The organic phase was washed with water (3 × 50 mL). The organic phase was then concentrated under reduced pressure. The crude product was purified by silica gel column chromatography (1:9 to 1:4 EtOAc/hexanes). Related fractions were combined, and the solvent was removed under reduced pressure. The mixture was diluted with hexane and kept in a freezer overnight. The yellow solid was filtered by vacuum filtration and dried under vacuum. **Yield:** 83% (3.25 g). **R*f***: 0.30 (15% EtOAc : Hexanes on silica gel). **Mp**: 113 - 116 °C. **^{1}H NMR (500 MHz, $CDCl_3$) δ (ppm)**: 7.72 (d, *J* = 7.7 Hz, 2H), 7.28 (d, *J* = 7.7 Hz, 1H), 7.12 – 7.18 (m, 2H), 6.94 – 6.95 (m, 2H), 6.88 (d, *J* = 7.9 Hz, 3H), 6.74 – 6.78 (m, 2H), 3.76 (s, 3H), 3.48 (s, 6H). **^{13}C NMR (126 MHz, $CDCl_3$) δ (ppm)**: 167.8, 155.5, 147.8, 144.7, 140.3, 133.8, 132.5, 128.9, 125.0(4), 124.9(5), 121.5, 121.0, 114.3, 55.6, 52.3. **GC-MS [M]$^+$** Retention time : 11.280; Calculated mass for $C_{23}H_{21}NO_5$: 391.42; Found: 391.15.

3-Hydroxyquinolino[3,2,1-*de*]acridine-5,9-dione (2)

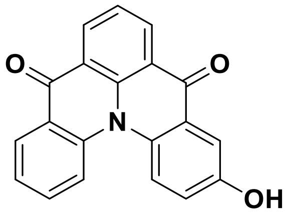

Compound **1** (3.00 g, 1 equiv) was dispersed in EtOH/H_2O (15 mL:15 mL) and mixed with NaOH (1.53 g, 5 equiv). The reaction mixture was heated to reflux for 3 h. After cooling to rt, the pH was adjusted to 2–3 by dropwise addition of dilute HCl. The resultant oily solid was kept in the fridge for 30 min and then sonicated for 60 min. The formed solid was then isolated by vacuum filtration and washed with water. Product was dried under vacuum. The diacid obtained was added to dry DCM (40 mL), oxalyl chloride (1.16 mL, 13.2 mmol, 2 equiv) in a two-necked flask fitted with condenser under nitrogen. To this mixture, 3 drops of dry DMF were added. After refluxing for 3 h, the reaction flask was cooled to rt, and $AlCl_3$ (7.04 g, 52.8 mmol, 8 equiv) was added in portions under a positive N_2 flow. It was heated to reflux for 20 h. After cooled to rt, the reaction was quenched by slow addition of water (50 mL) and THF (50 mL) was added to dissolve the product. The layers are separated, and the aqueous layer was washed with more DCM (5 × 100 mL). The organic fractions were combined and concentrated under reduced pressure. The orange precipitate formed was washed with acetone and DCM and dried under vacuum. **Yield:** 67% (1.40 g). **R*f***: 0.23 (2:3 EtOAc/hexanes on silica gel). **Mp**: 360–364 °C. **^{1}H NMR (500 MHz, DMSO-d$_6$) δ (ppm)**: 10.12 (s, 1H), 8.63 (d, *J* = 7.8, 0.8 Hz, 2H), 8.33 (dd, *J* = 7.8, 1.5 Hz, 1H), 8.17 (d, *J* = 8.5 Hz, 1H), 8.06 (d, *J* = 9.2 Hz, 1H), 7.83 (td, *J* = 7.3, 1.5 Hz, 1H), 7.72 (t, *J* = 7.6 Hz, 1H), 7.63 (d, *J* = 3.0 Hz, 1H), 7.55 (t, *J* = 7.3 Hz, 1H), 7.28 (dd, *J* = 9.2, 3.0 Hz, 1H). **^{13}C NMR (126 MHz, DMSO-d$_6$) δ (ppm)**: 177.6, 177.5, 154.9, 139.6, 138.3, 133.3, 132.4, 132.2, 127.0, 126.7, 125.4, 124.9, 123.4, 122.8, 122.7, 122.5, 121.9, 120.7, 109.9. **HR-MS [M-H]** Calculated: ($C_{20}H_{10}NO_3$) 312.0661; Found: 312.0669.

3-(4-Bromobutoxy)quinolino[3,2,1-*de*]acridine-5,9-dione (3)

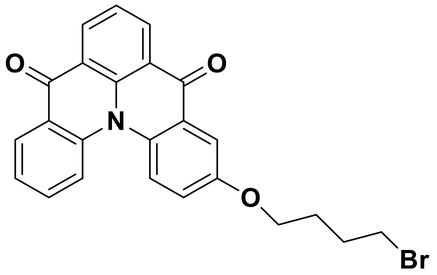

Compound **2** (1.25 g, 3.99 mmol, 1 equiv) and NaO*t*-Bu (1.15 g, 12.0 mmol, 3 equiv) was added to a Schlenk tube under N_2 flow. Dry THF (20 mL) was injected and stirred for 30 minutes. To this solution, 1,4-dibromobutane (4.73 mL, 39.9 mmol, 10 equiv) was added. The reaction was stirred at 65 °C for

16 h. The mixture was cooled to rt, diluted with DCM (100 mL). The DCM layer was then washed with water (3 × 50 mL). The organic phase was concentrated under reduced pressure. The compound was further purified by silica gel column chromatography (3:7 EtOAc/hexane). The fractions were combined and concentrated under reduced pressure. The yellow solid was then filtered and dried under vacuum. Product was obtained as a yellow solid. **Yield:** 40 % (0.71 g). **R*f***: 0.47 (2:3 EtOAc/hexanes on silica gel). **Mp**: 158 – 161 °C. **^{1}H NMR (500 MHz, CDCl$_3$) δ (ppm)**: 8.80-8.73 (m, 2H), 8.51 (dd, *J* = 7.9, 1.7 Hz, 1H), 8.09 (dd, *J* = 13.4, 8.9 Hz, 2H), 7.87 (d, *J* = 3.1 Hz, 1H), 7.71 (ddd, *J* = 8.7, 7.1, 1.7 Hz, 1H), 7.66 (t, *J* = 7.6 Hz, 1H), 7.49 (t, *J* = 7.9 Hz, 1H), 7.31 - 7.28 (m, 2H), 4.21 (t, *J* = 6.0 Hz, 1H), 3.55 (t, *J* = 6.5 Hz, 2H), 2.19 - 2.11 (m, 2H), 2.10 - 2.02 (m, 2H). **^{13}C NMR (126 MHz, CDCl$_3$) δ (ppm)**: 178.5, 178.3, 156.1, 139.8, 138.8, 133.8, 133.1, 132.7, 127.8, 127.4, 126.2, 125.0, 123.3, 122.9, 122.4, 122.0, 120.1, 108.3, 67.7, 33.4, 29.5, 27.9. **HR-MS [M+]** Calculated: ($C_{24}H_{18}BrNO_3$) 448.0470; Found: 448.0540.

3-(4-((5,9-Dioxo-5,9-dihydroquinolino[3,2,1-*de*]acridin-3-yl)oxy)butyl)-1-methyl-1*H*-imidazol-3-ium hexafluorophosphate(V) (DiKTa-OBuIm)

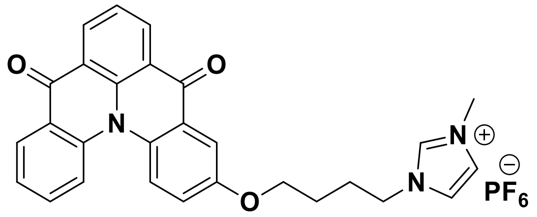

Compound **3** (0.7 g, 2 mmol, 1 equiv) and 1-methylimidazole (4 mL, 80 mmol, 40 equiv) was added to a Schlenk tube under nitrogen. The reaction was stirred at 100 °C for 2 h. After cooling to rt, ammonium hexafluorophosphate solution (3.0 g/ 3 mL) was added dropwise to the mixture (3 mL) and stirred for 15 min. The mixture was diluted with cold water (50 mL). The yellowish-orange precipitate was filtered under vacuum, washed with water (100 mL) and then dried under vacuum. The product was obtained as a yellow solid. **Yield:** 85% (0.76 g). **Mp**: 105 - 108 °C. **^{1}H NMR (500 MHz, DMSO-d$_6$) δ (ppm)**: 9.16 (d, *J* = 1.7 Hz, 2H), 8.61 (dq, *J* = 7.7, 1.7 Hz, 2H), 8.32 (dd, *J* = 7.9, 1.7 Hz, 2H), 8.14 - 8.08 (m, 1H), 7.87-7.79 (m, 1H), 7.74 - 7.69 (m, 3H), 7.56 (t, *J* = 7.5 Hz, 1H), 7.40 (dd, *J* = 9.3, 3.1 Hz, 1H), 4.30 (t, *J* = 7.1 Hz, 2H), 4.20 (t, *J* = 6.3 Hz, 2H), 3.87 (s, 3H), 2.09 - 1.98 (m, 2H), 1.88 - 1.75 (m, 2H). **^{13}C NMR (126 MHz, DMSO-d$_6$) δ (ppm)**: 178.0, 177.7, 156.0, 139.8, 138.7, 133.8, 132.7, 127.1,

125.8, 125.5, 124.1, 124.0, 123.2, 122.7, 122.4, 121.0, 108.2, 67.8, 48.9, 36.2, 26.7, 25.6. **^{19}F {^{1}H} NMR (471 MHz, DMSO-d$_6$) δ (ppm):** -70.8 (d, *J* = 710.5 Hz). **^{31}P {^{1}H} NMR (202 MHz, DMSO-d$_6$) δ (ppm):** -144.2 (hept, *J* = 711.4 Hz). **HR-MS [M+]** Calculated: ($C_{23}H_{21}NO_5Na$) 450.1812; Found: 450.1825.

3-((4-Methoxyphenyl)(phenyl)amino)quinolino[3,2,1-*de*]acridine-5,9-dione (4)

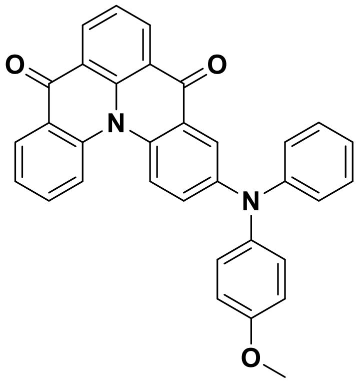

Br-DiKTa [3] (1.00 g, 2.66 mmol, 1 equiv), 4-methoxy-*N*-phenylaniline (0.56 g, 2.79 mmol, 1.05 equiv), NaO*t*-Bu (0.51 g, 5.32 mmol, 2 equiv), tri-*tert*-butylphosphonium tetrafluoroborate (0.05 g, 0.19 mmol, 0.07 equiv) and $Pd_2(dba)_3$ (0.048 g, 0.053 mmol, 0.02 equiv) were added in a Schlenk tube under nitrogen. Dry toluene (12 mL) was injected tube and the mixture was heated to 110 °C. After 24 h, the reaction mixture was cooled to rt, extracted with DCM (200 mL), and washed with water (3 × 50 mL). The desired product was obtained as a red solid after column chromatography (1:9 EtOAc/hexanes). **Yield:** 78% (1.02 g). **R*f***: 0.5 (2:3 EtOAc : Hexanes on silica gel). **Mp**: 217 - 219 °C. **^{1}H NMR (500 MHz, $CDCl_3$) δ (ppm):** 8.76 (dd, *J* = 7.7, 1.7 Hz, 1H), 8.72 (dd, *J* = 7.6, 1.7 Hz, 1H), 8.51 (dd, *J* = 7.9, 1.7 Hz, 1H), 8.16 (d, *J* = 8.6 Hz, 1H), 8.04 (d, *J* = 2.8 Hz, 1H), 7.98 (d, *J* = 9.2 Hz, 1H), 7.73-7.67 (m, 1H), 7.63 (t, *J* = 7.6 Hz, 1H), 7.49 (t, *J* = 7.5 Hz, 1H), 7.39 (dd, *J* = 9.2, 2.9 Hz, 1H), 7.34 - 7.30 (m, 2H), 7.19 - 7.13 (m, 4H), 7.08 (tt, *J* = 7.4, 1.2 Hz, 1H), 6.95 - 6.91 (m, 2H), 3.86 (s, 3H). **^{13}C NMR (126 MHz, $CDCl_3$) δ (ppm):** 178.5, 178.3, 139.8, 138.8, 133.0, 132.8, 132.6, 129.5, 127.8, 126.3, 124.8, 123.3, 121.3, 120.3, 117.5, 115.1, 55.5. **HR-MS [M+]** Calculated: ($C_{33}H_{22}N_2O_3$) 495.1630; Found: 495.1634.

3-((4-Hydroxyphenyl)(phenyl)amino)quinolino[3,2,1-*de*]acridine-5,9-dione (5)

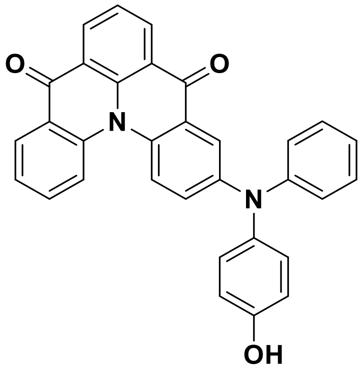

Compound **4** (0.80 g, 1.62 mmol, 1 equiv) was dissolved in dry DCM (10 mL) in a Schlenk flask under nitrogen cover. BBr_3 in DCM solution (8.1 mL, 8.09 mmol, 5 equiv) was dropwise added to the solution at −78 °C. After 2 h water was added slowly, and the crude reaction was extracted with DCM (100 mL). The compound was further purified by silica gel column chromatography (1:1 EtOAc/hexanes). The product was obtained as a purple solid. **Yield:** 65% (0.50 g). **R*f***: 0.23 (2:3 EtOAc : Hexanes on silica gel). **Mp**: 269 - 272 °C. **^{1}H NMR (500 MHz, DMSO-d$_6$) δ (ppm):** 9.59 (s, 1H), 8.59 (dd, *J* = 7.5, 1.8 Hz, 1H), 8.54 (dd, *J* = 7.6, 1.8 Hz, 1H), 8.30 (d, *J* = 7.9 Hz, 1H), 8.16 (d, *J* = 8.4 Hz, 1H), 8.02 (dd, *J* = 9.7, 4.5 Hz, 1H), 7.81 (t, *J* = 7.8 Hz, 1H), 7.73 - 7.66 (m, 1H), 7.63 (t, *J* = 3.0 Hz, 1H), 7.54 (t, *J* = 7.5 Hz, 1H), 7.39 - 7.30 (m, 3H), 7.15 - 7.04 (m, 5H), 6.84 (d, *J* = 8.7 Hz, 2H). **^{13}C NMR (126 MHz, DMSO-d$_6$) δ (ppm):** 177.9, 177.7, 155.7, 147.3, 145.7, 139.8, 139.7, 137.8, 133.7, 133.3, 132.8, 132.6, 130.1, 129.8, 127.1, 126.9, 125.7, 125.5, 123.9, 123.6, 123.2, 122.8, 122.7, 121.3, 117.1, 115.1 **HR-MS [M]$^+$** Calculated: ($C_{32}H_{20}N_2O_3$) 480.1473; Found: 480.1444.

3-((4-(4-Bromobutoxy)phenyl)(phenyl)amino)quinolino[3,2,1-*de*]acridine-5,9-dione (6)

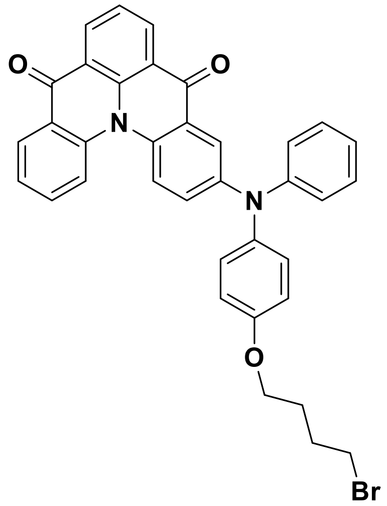

Compound **5** (0.650 g, 1.31 mmol, 1 equiv) and NaO*t*-Bu (0.379 g, 3.94 mmol, 3 equiv) were added to a dry Schlenk tube under a positive N_2 flow. Then dry THF (20 mL) and 1,4-dibromobutane (1.6 mL, 13.1 mmol, 10 equiv) were sequentially added to the mixture with stirring. The reaction was heated to 65 °C for 12 h. The mixture was extracted with DCM (100 mL) and washed with water (3 × 100 mL). The organic layer was concentrated under reduced pressure. The product was purified by silica gel column chromatography (1:9 EtOAc/hexanes on silica gel) and obtained as a red solid. **Yield:** 58% (0.47 g). **Rf**: 0.6 (2:3 EtOAc : Hexanes on silica gel). **Mp**: 121 – 125 °C. **^{1}H NMR (500 MHz, CDCl$_3$) δ (ppm):** 8.76 (dd, *J* = 7.6, 1.7 Hz, 1H), 8.72 (dd, *J* = 7.7, 1.7 Hz, 1H), 8.51 (dd, *J* = 7.9, 1.7 Hz, 1H), 8.16 (d, *J* = 8.5 Hz, 1H), 8.03 (d, *J* = 2.8 Hz, 1H), 7.98 (d, *J* = 9.2 Hz, 1H), 7.70 (t, *J* = 8.7 Hz, 1H), 7.64 (t, *J* = 7.7 Hz, 1H), 7.49 (t, *J* = 7.5 Hz, 1H), 7.39 (dd, *J* = 9.2, 2.9 Hz, 1H), 7.33 - 7.30 (m, 2H), 7.18 - 7.13 (m, 4H), 7.08 (tt, *J* = 7.3, 1.2 Hz, 1H), 6.94 - 6.88 (m, 2H), 4.04 (t, *J* = 6.0 Hz, 2H), 3.54 (t, *J* = 6.6 Hz, 2H), 2.17 - 2.09 (m, 2H), 2.03 - 1.96 (m, 2H). **^{13}C NMR (126 MHz, CDCl$_3$) δ (ppm):** 178.5, 178.3, 156.2, 147.1, 145.7, 133.6, 133.1, 132.6, 129.6, 127.7, 124.9, 123.8, 123.3, 121.4, 120.3, 117.6, 115.7, 67.1, 33.5, 31.6, 29.4, 27.9, 27.6, 14.1. **HR-MS [M]$^+$** ($C_{36}H_{27}BrN_2O_3$) Calculated: 615.5300; Found:617.1263

1-(4-(4-((5,9-Dioxo-5,9-dihydroquinolino[3,2,1-*de*]acridin-3-yl)(phenyl)amino)phenoxy)butyl)-3-methyl-1*H*-imidazol-3-ium hexafluorophosphate(V) (DiKTa-DPA-OBuIm)

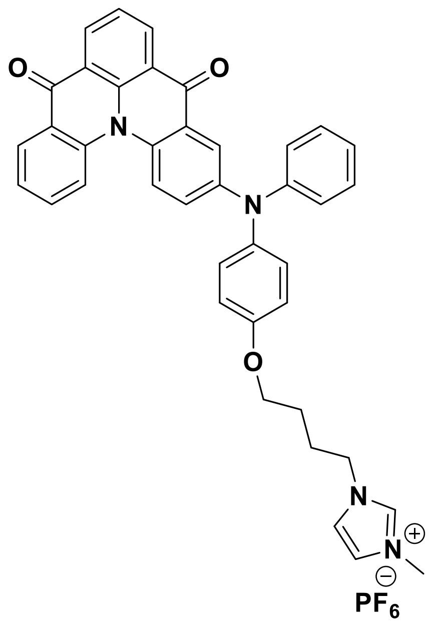

Compound **6** (0.43 g, 0.7 mmol, 1 equiv) and 1-methylimidazole (1 mL, 13 mmol, 18.6 equiv) was added to a Schlenk tube under nitrogen. The mixture was stirred for 2 h at 100 °C. After cooling to rt, ammonium hexafluorophosphate solution (2 g in 2 mL) was added to the reaction mixture and the re-action mixture was stirred for 15 min. The product precipitated after addition of water (3 mL) and then placed in the fridge overnight. The red precipitate was filtered under vacuum, washed with water (100 mL) and dried under vacuum. The product was obtained as red solid. **Yield:** 93% (0.54 g). **Mp**: 239 - 241 °C. **^{1}H NMR (700 MHz, DMSO-d$_6$) δ (ppm):** δ 9.14 (s, 1H), 8.62 (d, *J* = 7.6 Hz, 1H), 8.56 (d, *J* = 7.6 Hz, 1H), 8.33 (d, *J* = 7.8 Hz, 1H), 8.18 (d, *J* = 8.6 Hz, 1H), 8.08 (d, *J* = 9.3 Hz, 1H), 7.88 – 7.76 (m, 2H), 7.73 – 7.68 (m, 2H), 7.65 (d, *J* = 2.7 Hz, 1H), 7.38-7.36 (m, 3H), 7.37 (t, *J* = 7.8 Hz, 2H), 7.17 (d, *J* = 8.8 Hz, 2H), 7.13-7.10 (m, 3H), 7.00 (d, *J* = 8.9 Hz, 2H), 4.27 (t, *J* = 7.1 Hz, 2H), 4.03 (t, *J* = 6.1 Hz, 2H), 3.86 (s, 3H), 1.98 (p, *J* = 7.3 Hz, 2H), 1.74 (p, *J* = 6.2 Hz, 2H). **^{13}C NMR (126 MHz, DMSO-d$_6$) δ (ppm):** 178.0, 156.4, 147.2, 145.5, 139.5, 137.0, 133.0, 132.7, 128.4, 124.1, 122.7, 116.3, 67.4, 48.7, 36.2, 26.8, 25.8. **^{19}F {^{1}H} NMR (471 MHz, DMSO-d$_6$) δ (ppm):** -70.1 (d, *J* = 710.5 Hz). **^{31}P {^{1}H} NMR (202 MHz, DMSO-d$_6$) δ (ppm):** -144.2 (hept, *J* = 711.4 Hz). **HR-MS [M]$^+$** ($C_{40}H_{33}N_4O_3$) Calculated: 617.2542; Found:617.2553

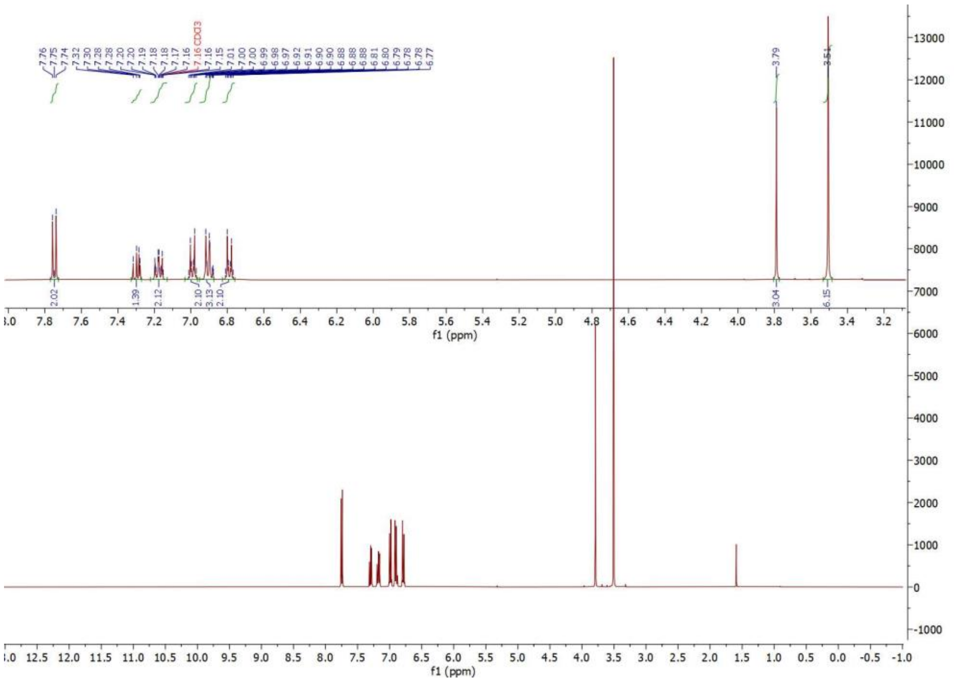

Figure S1. ^{1}HNMR spectrum of **1** in $CDCl_3$

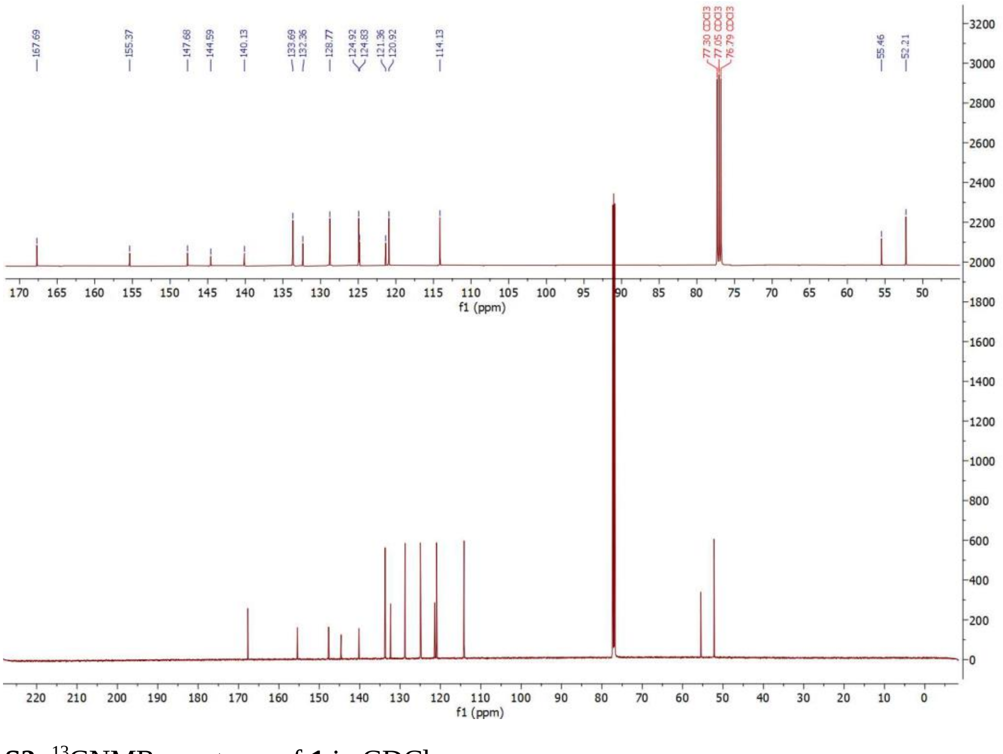

Figure S2. ^{13}CNMR spectrum of **1** in $CDCl_3$

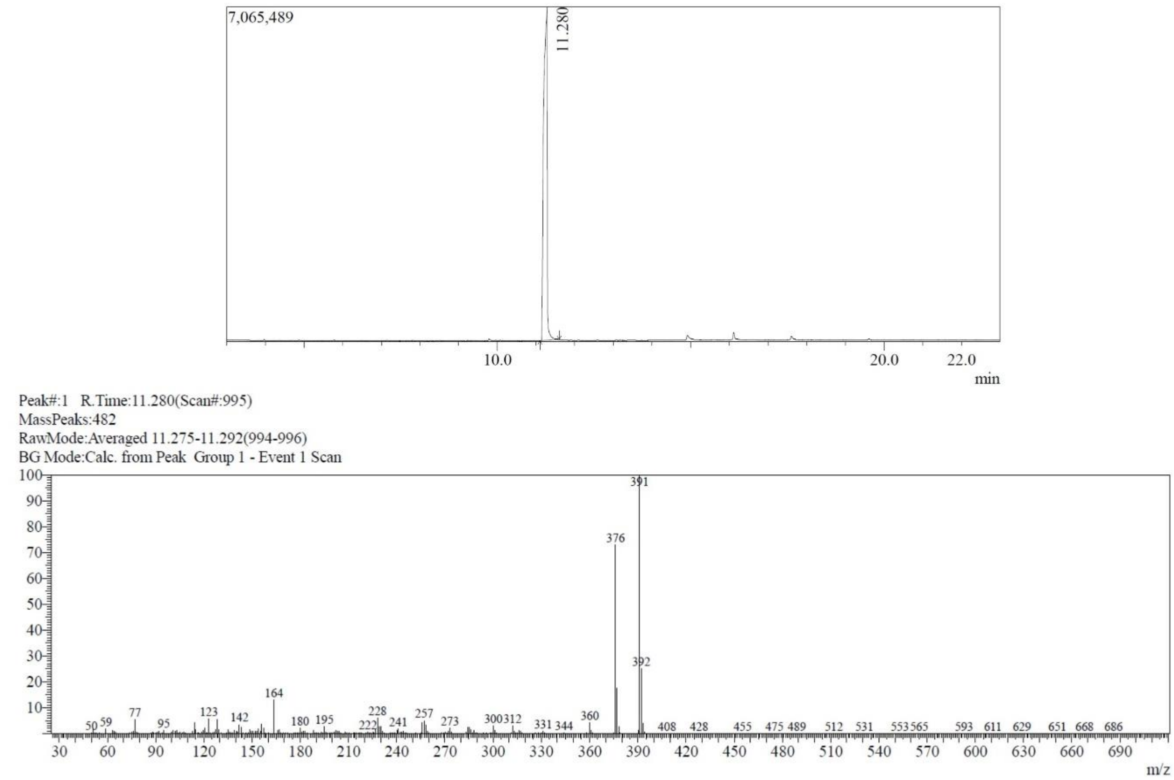

Figure S3. GCMS of **1**. Base m/z is 391.15.

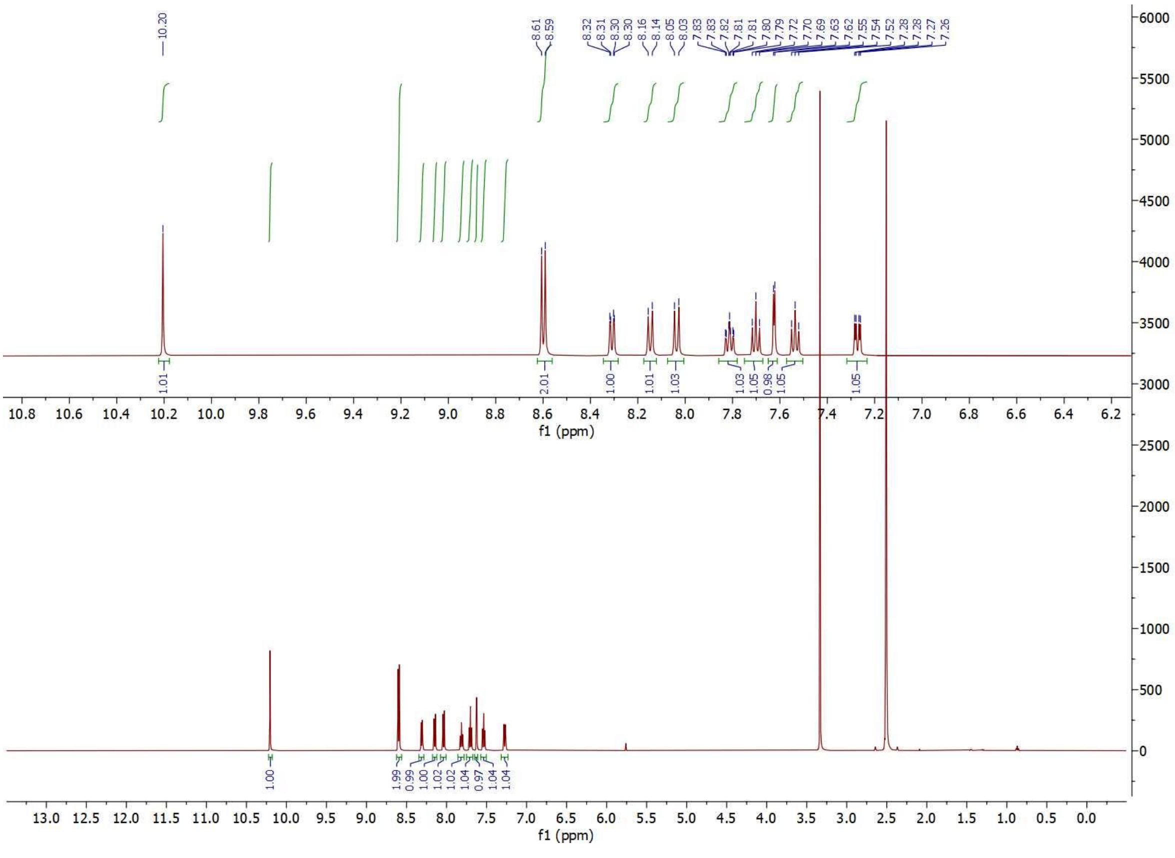

Figure S4. ^{1}HNMR spectrum of **2** in DMSO-d_6.

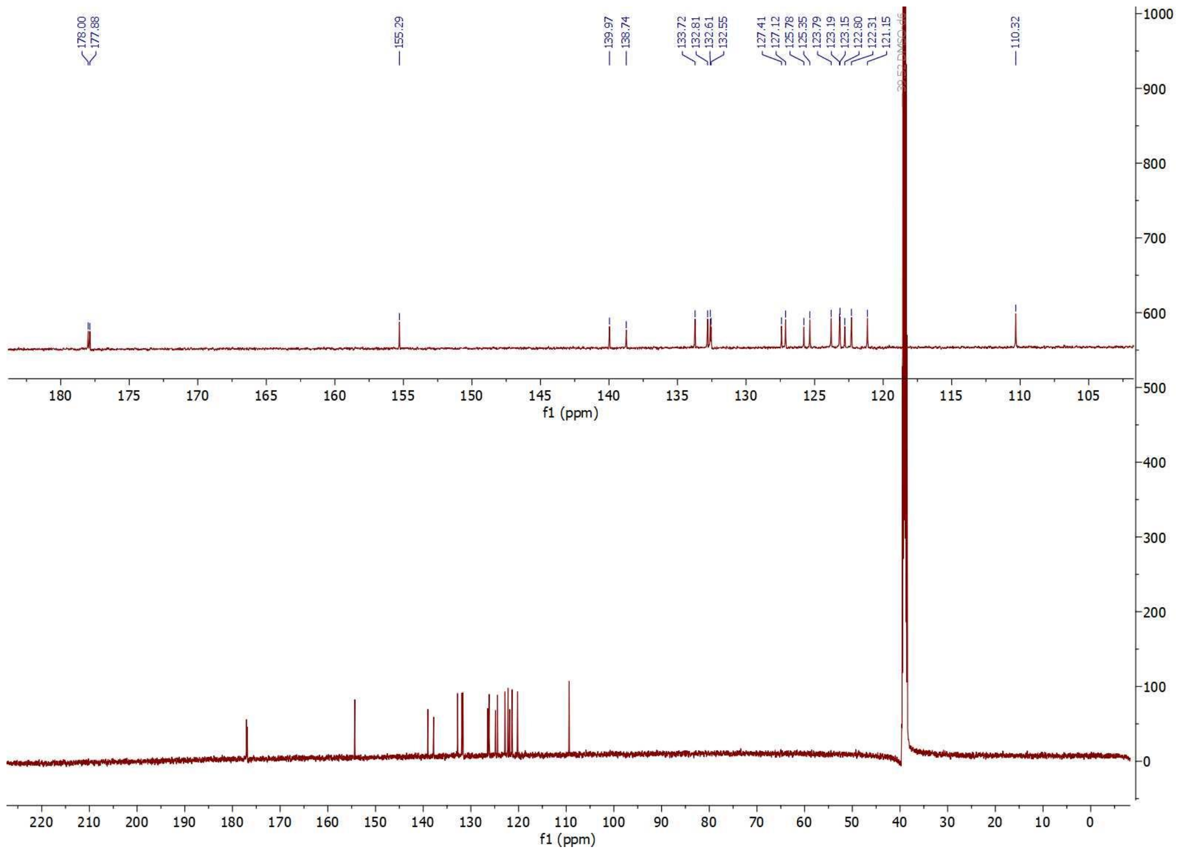

Figure S5. ^{13}CNMR spectrum of **2** in DMSO-d_6.

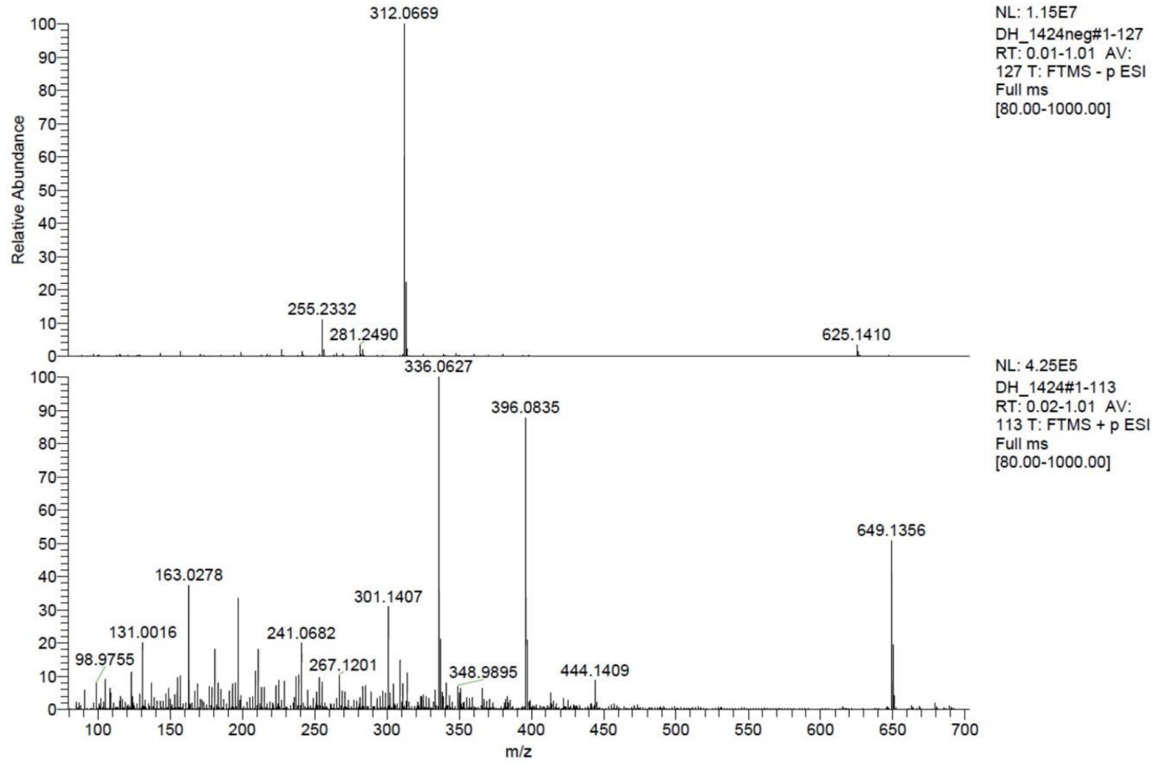

Figure S6. High resolution electrospray ionization mass spectrometry (ESI-HRMS) of **2**.

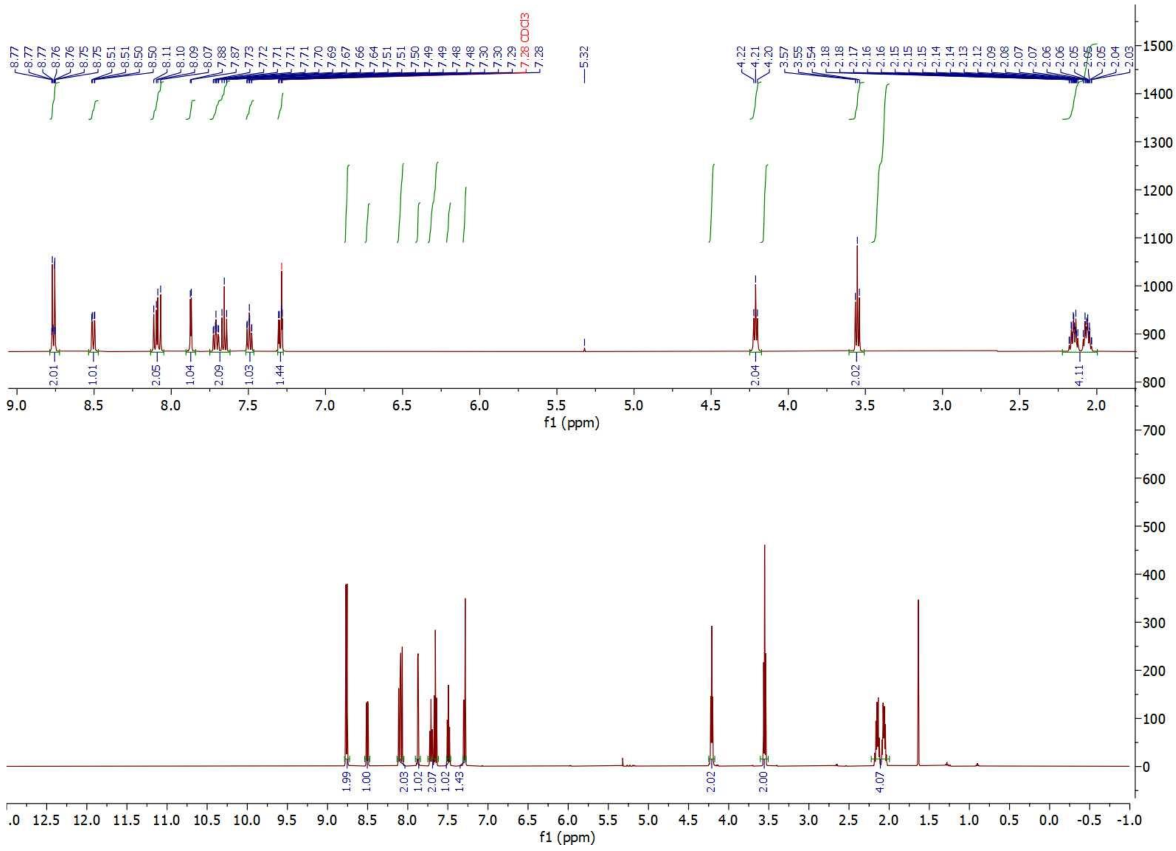

Figure S7. ^{1}HNMR spectrum of **3** in $CDCl_3$.

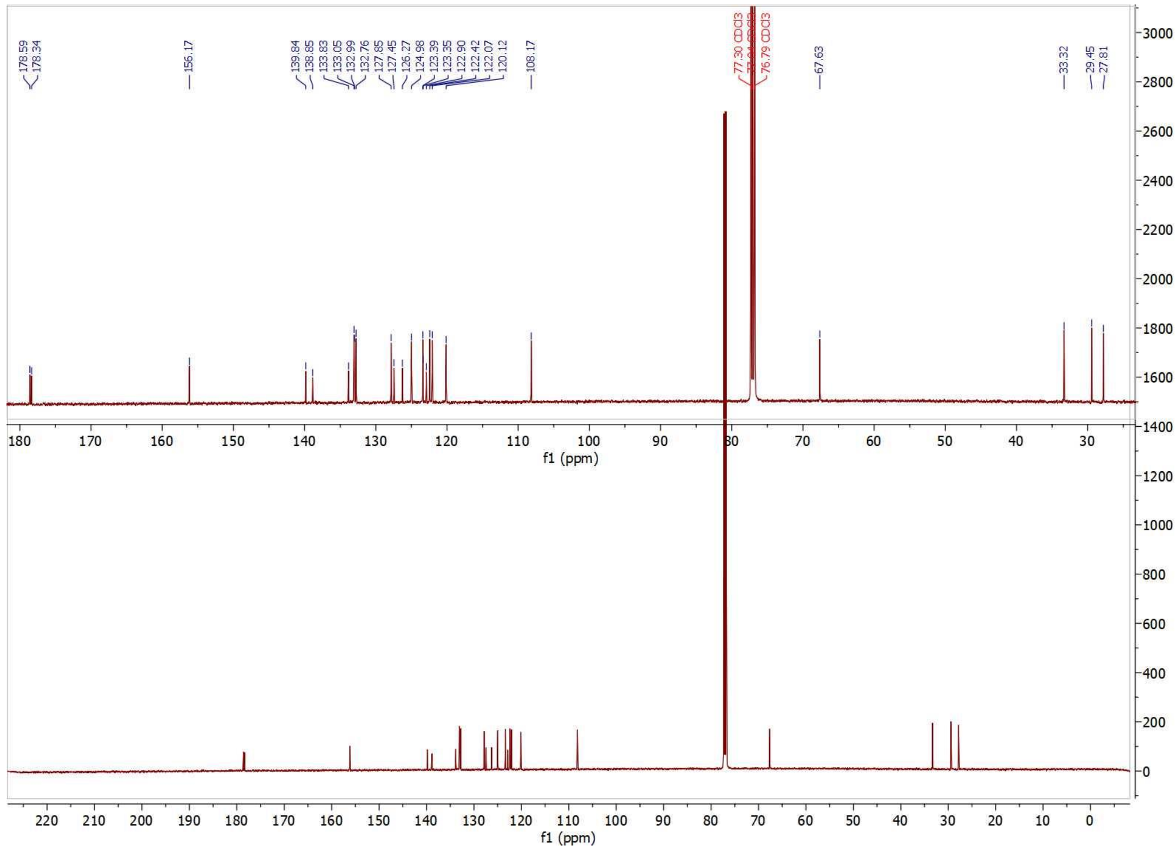

Figure S8. ^{13}CNMR spectrum of **3** in $CDCl_3$.

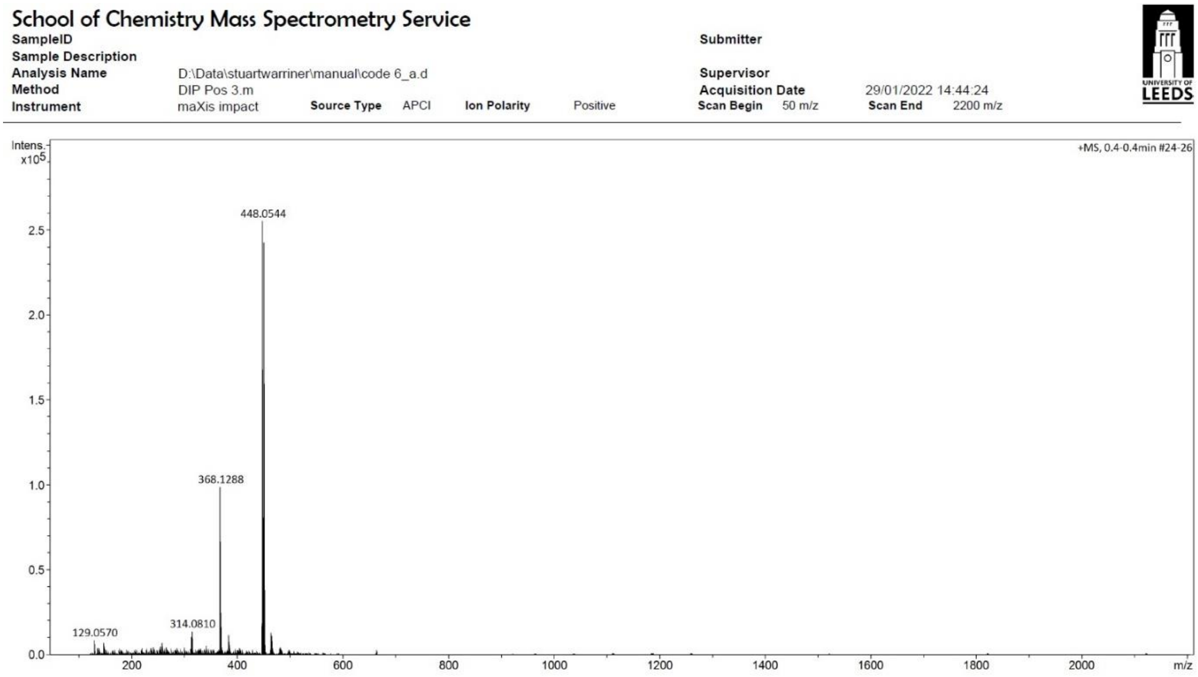

Figure S9. APCI HR-MS of **3**.

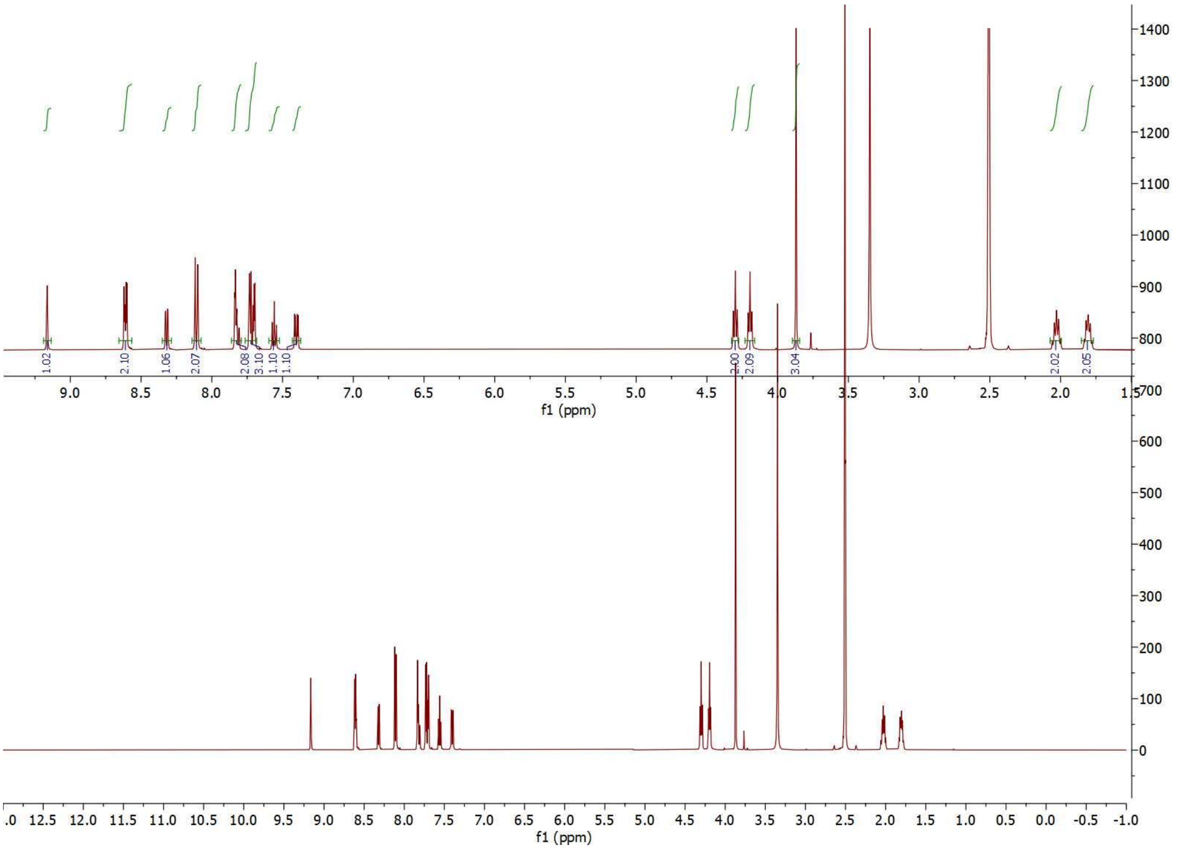

Figure S10. ^{1}HNMR spectrum of **DiKTa-OBuIm** in DMSO-d_6.

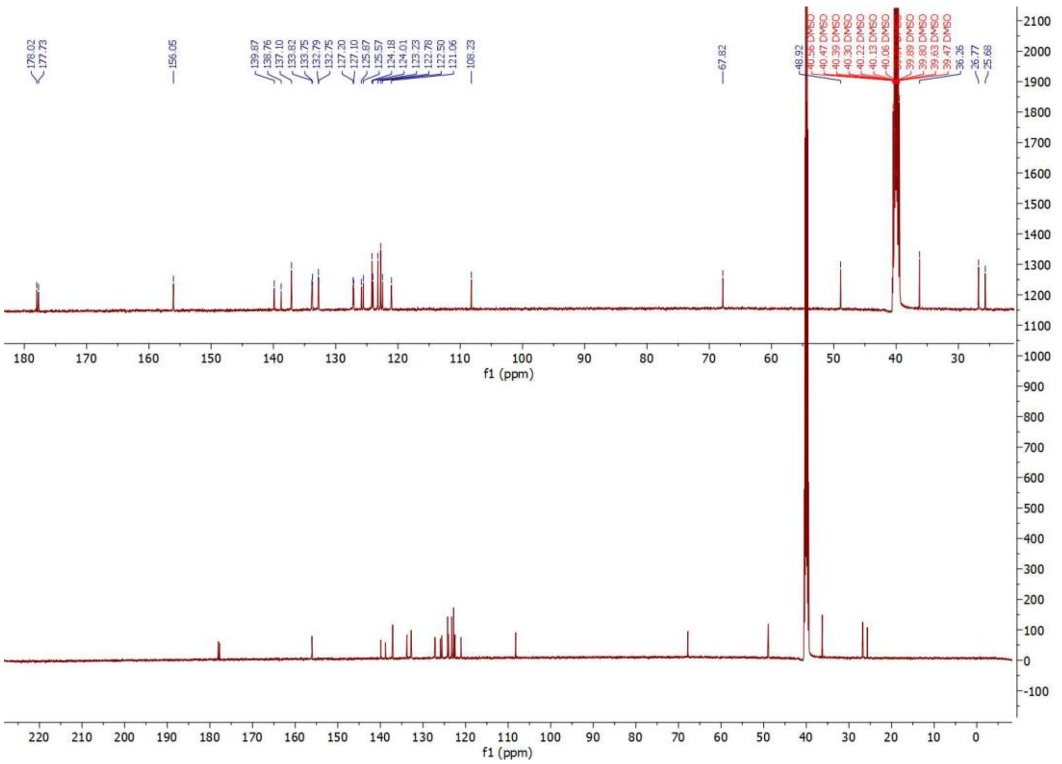

Figure S11. ^{13}CNMR spectrum of **DiKTa-OBuIm** in DMSO-d_6.

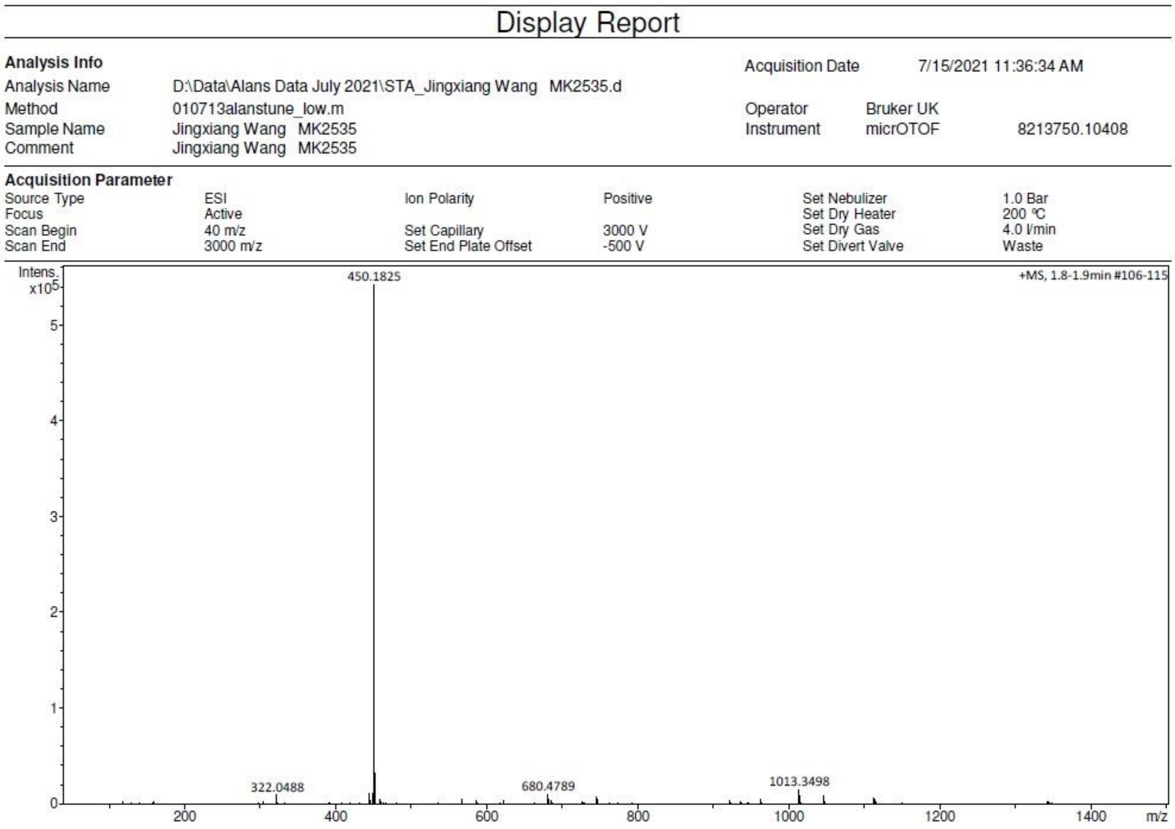

Figure S12. HRMS of **DiKTa-OBuIm**.

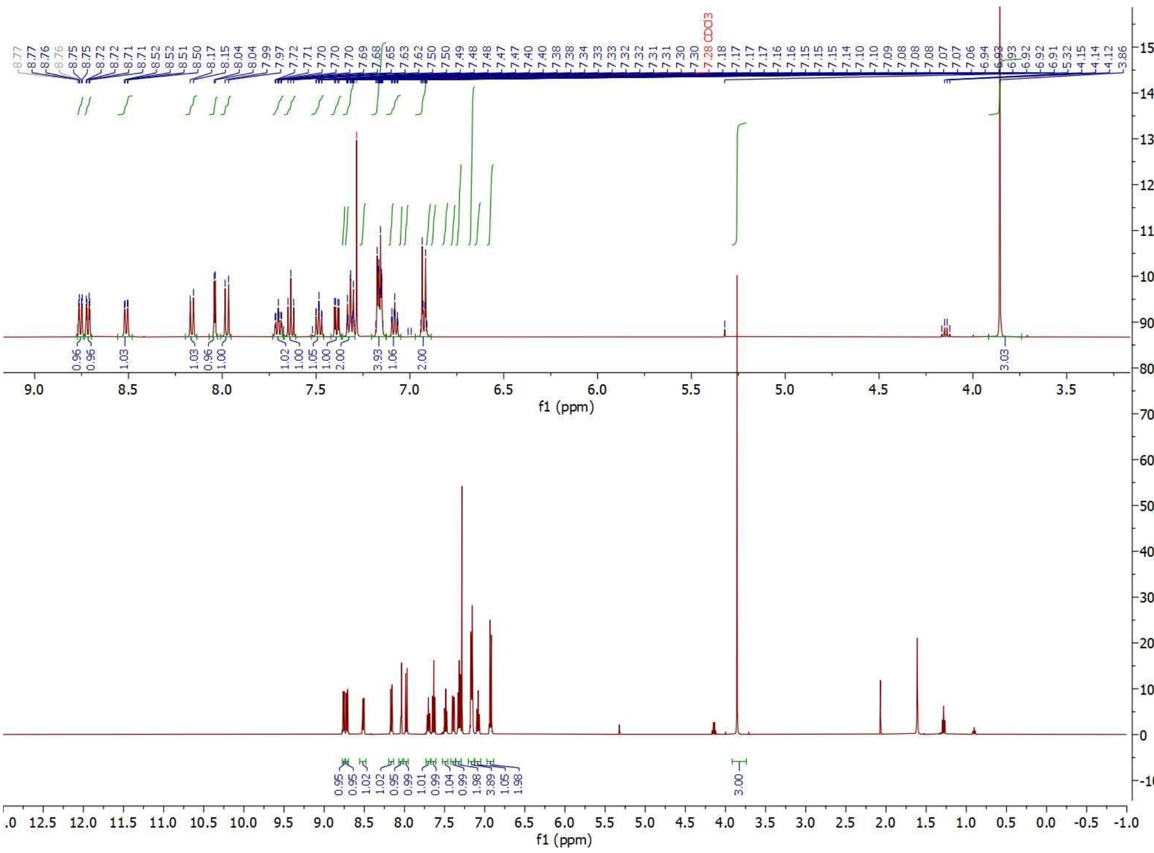

Figure S13. ^{1}HNMR spectrum of **4** in $CDCl_3$.

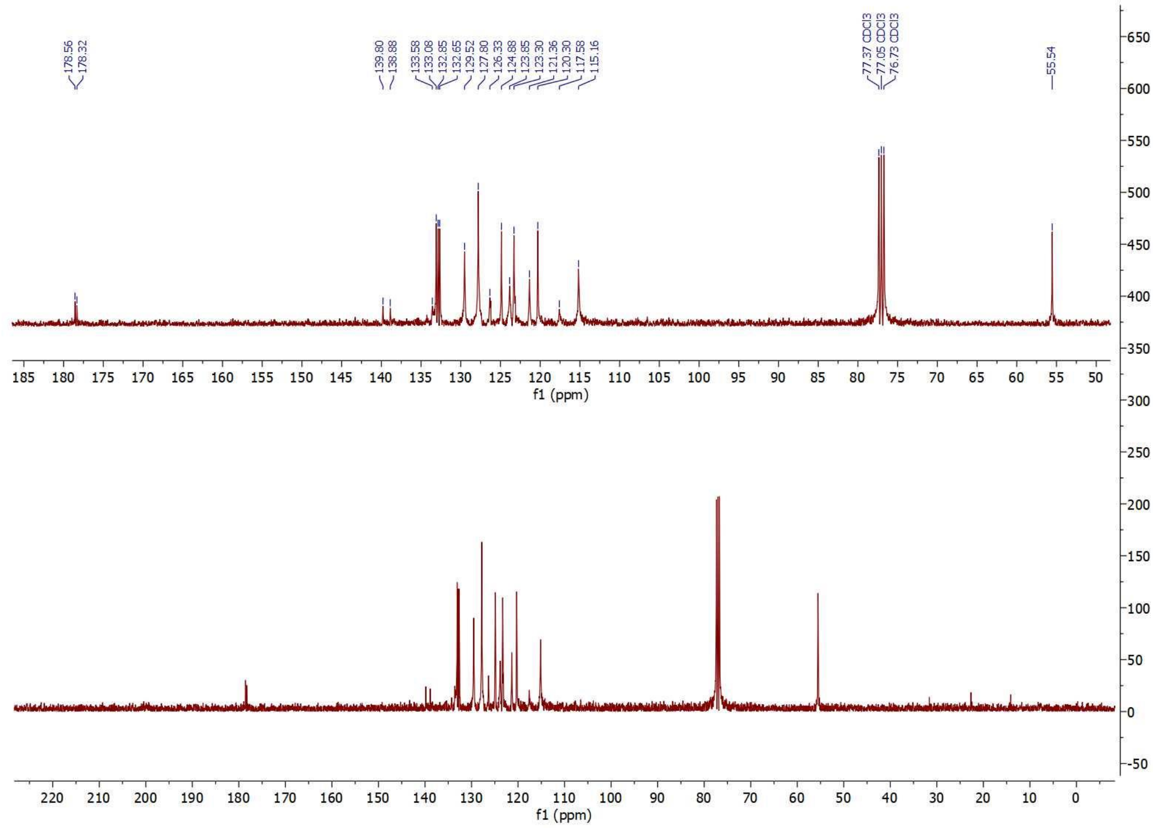

Figure S14. ^{13}CNMR spectrum of **4** in $CDCl_3$.

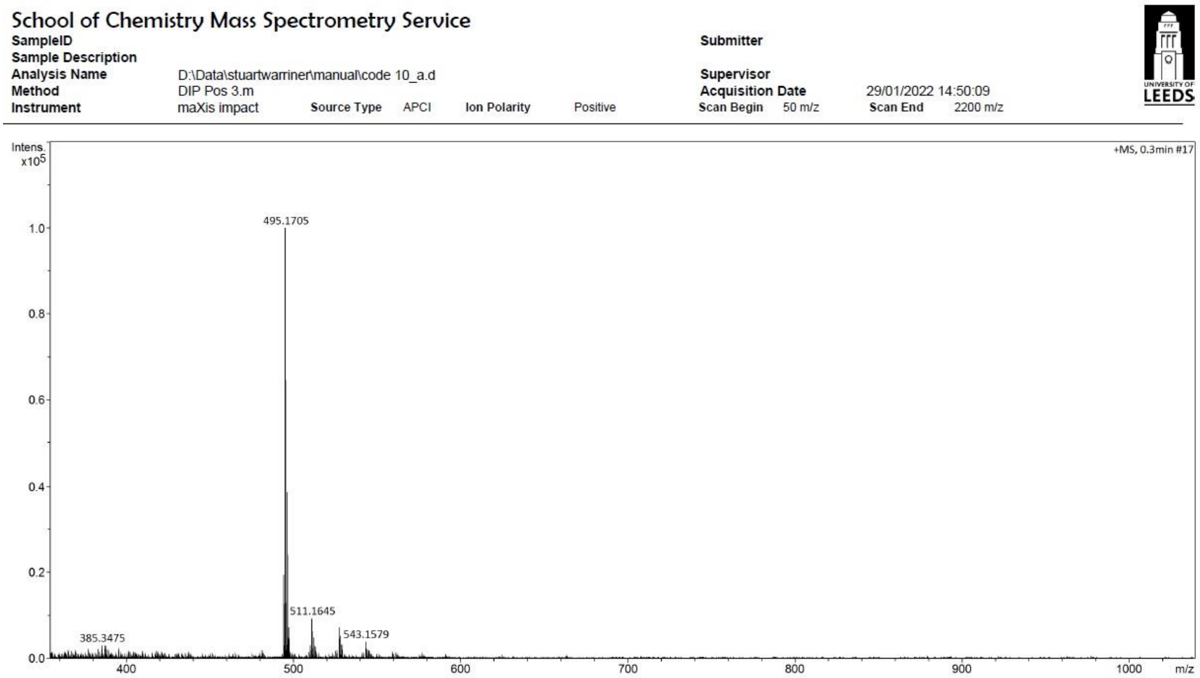

Figure S15. HRMS of **4**.

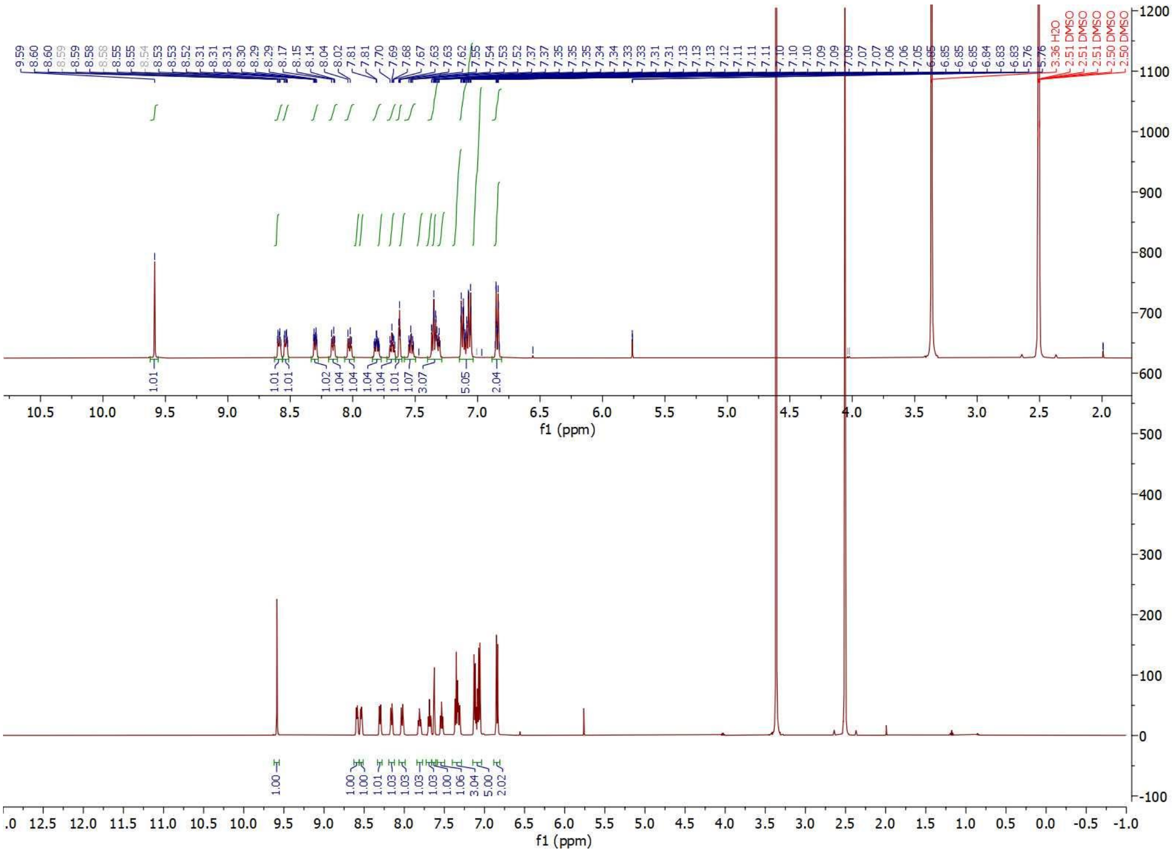

Figure S16. ^{1}HNMR spectrum of **5** in DMSO-d_6.

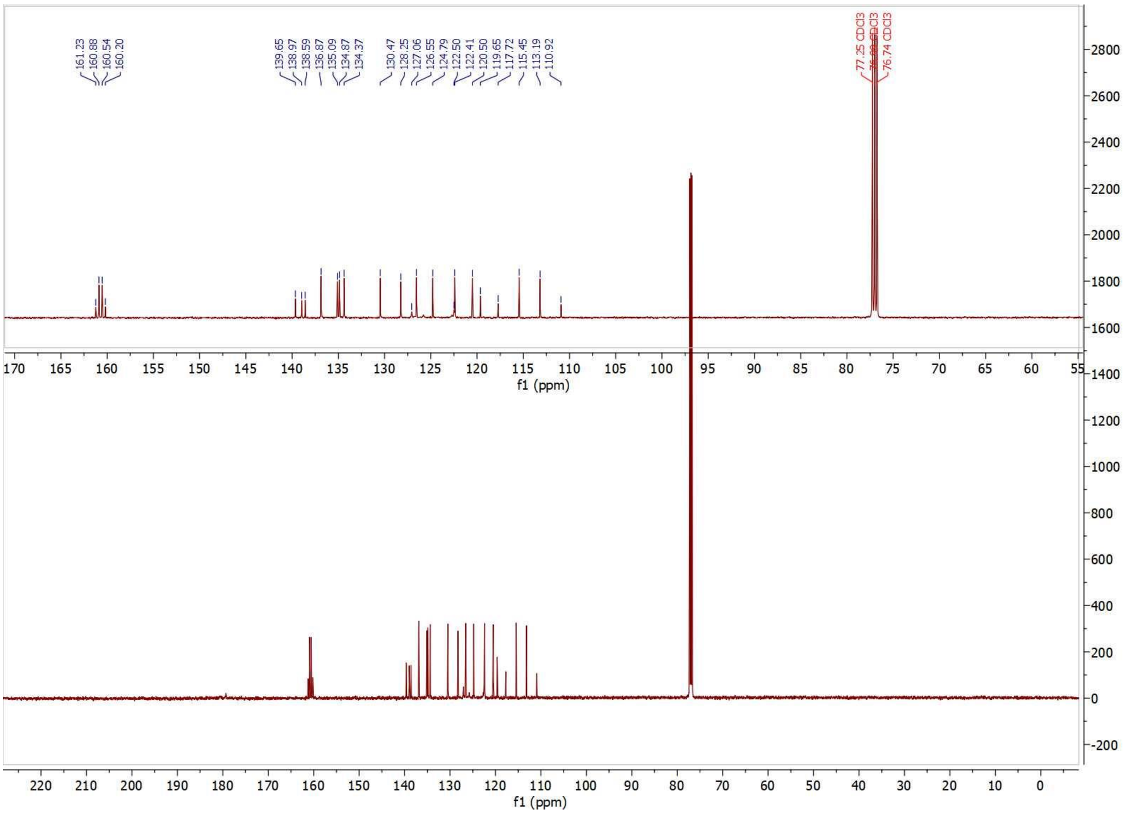

Figure S17. ^{13}CNMR spectrum of **5** in DMSO-d_6.

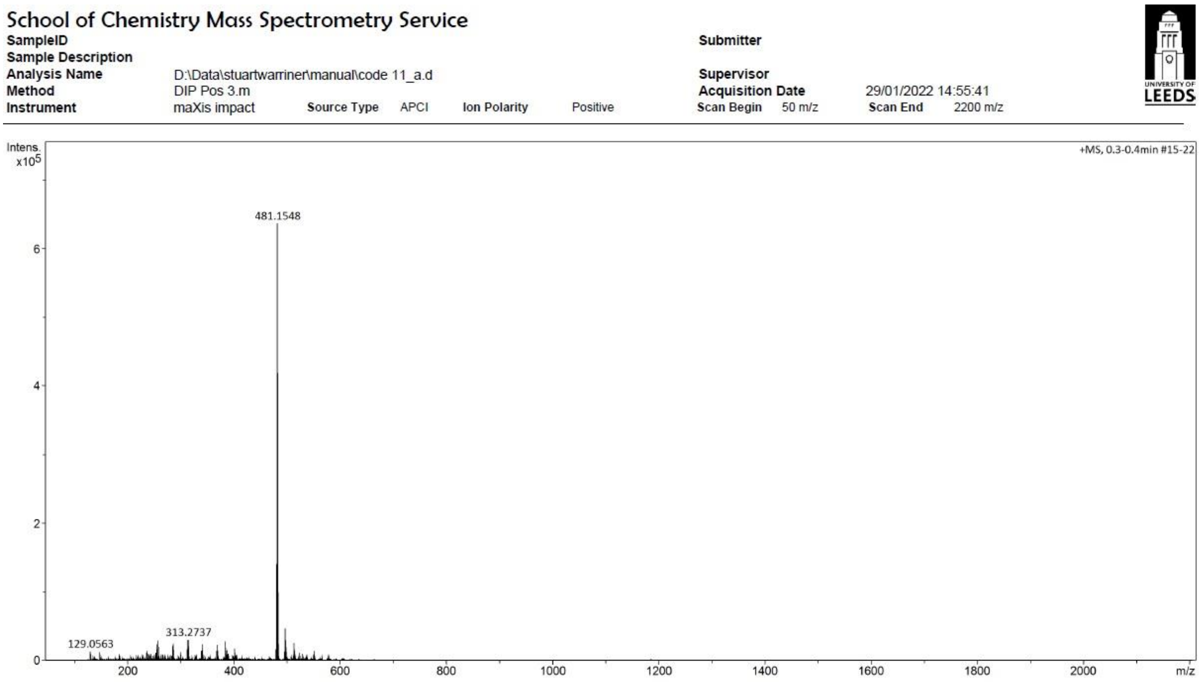

Figure S18. HRMS of **5**.

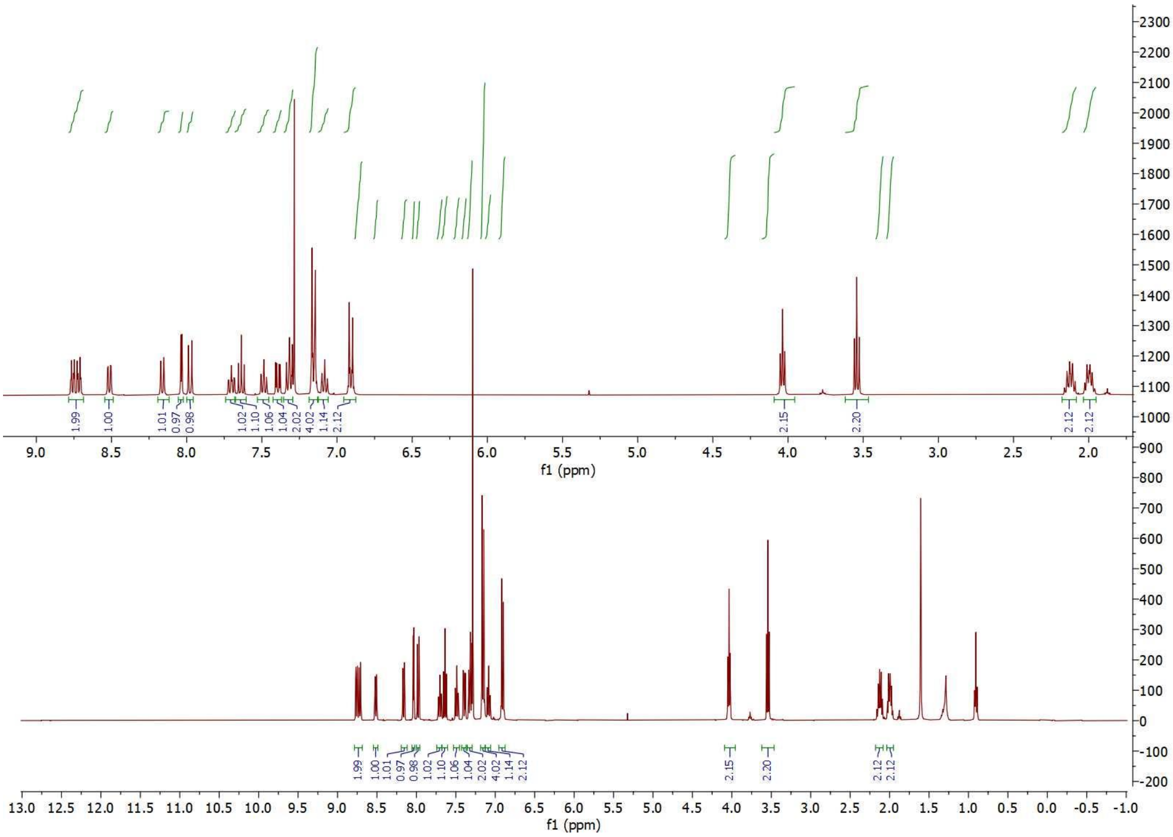

Figure S19. ^{1}HNMR spectrum of 6 in $CDCl_3$.

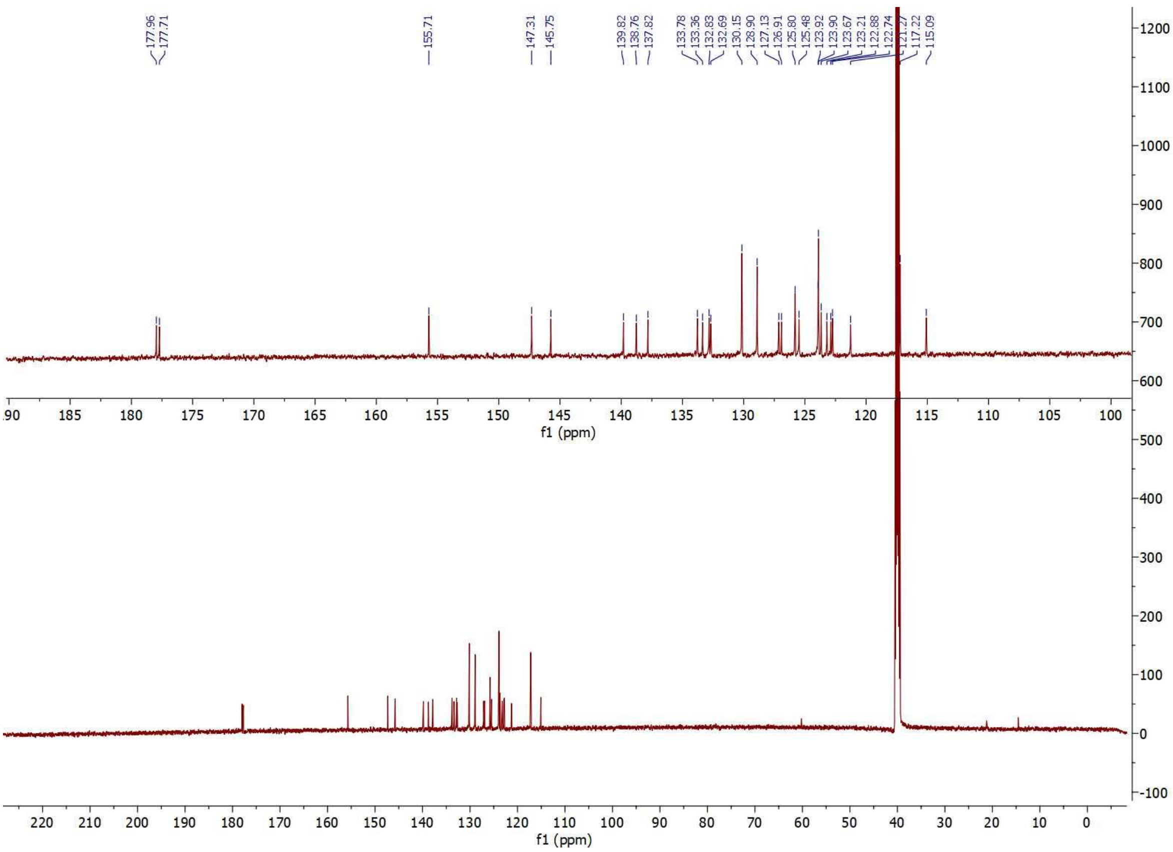

Figure S20. ^{13}CNMR spectrum of 6 in $CDCl_3$.

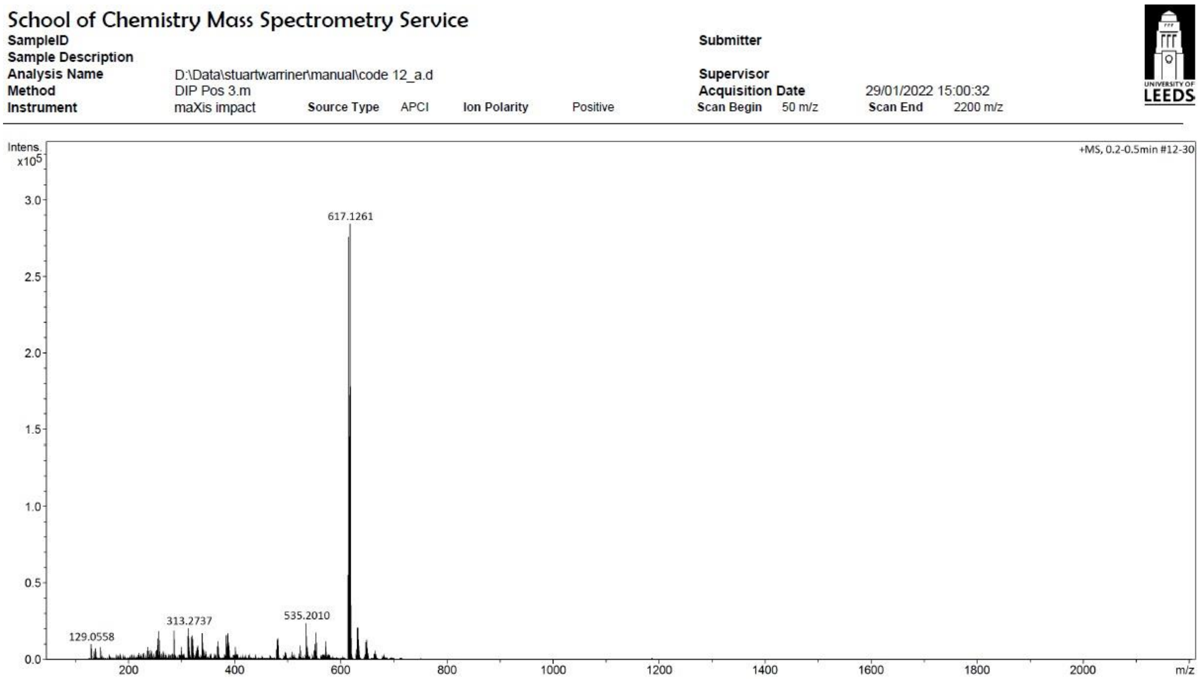

Figure S21. HR-MS of **6**.

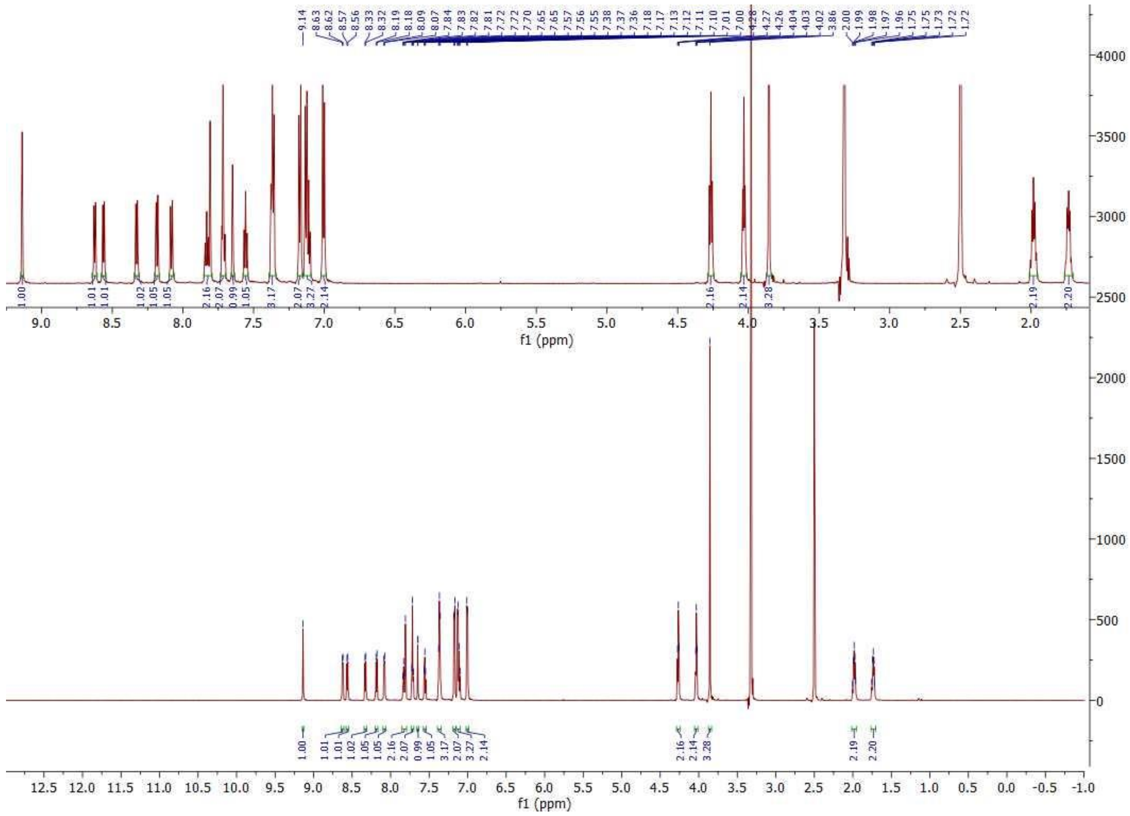

Figure S22. ^{1}HNMR spectrum of **DiKTa-DPA-OBuIm** in DMSO-d_6.

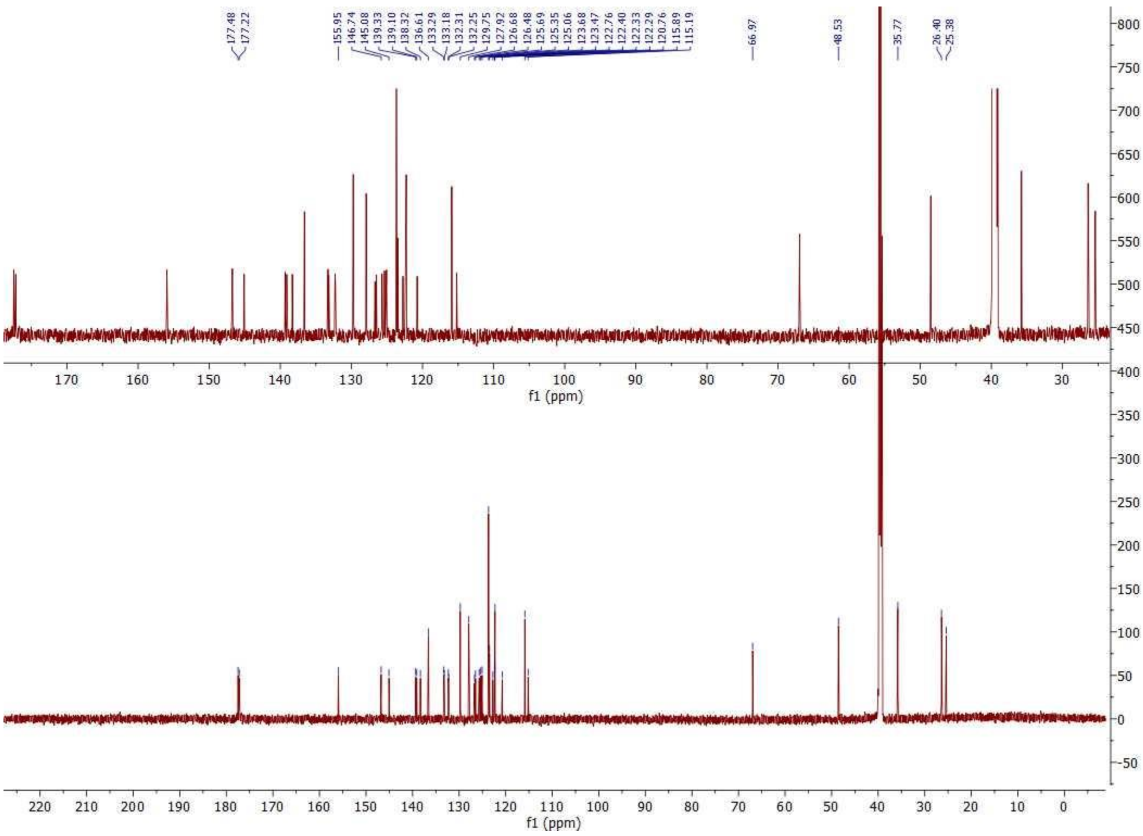

Figure S23. ^{13}CNMR spectrum of **DiKTa-DPA-OBuIm** in DMSO-d_6.

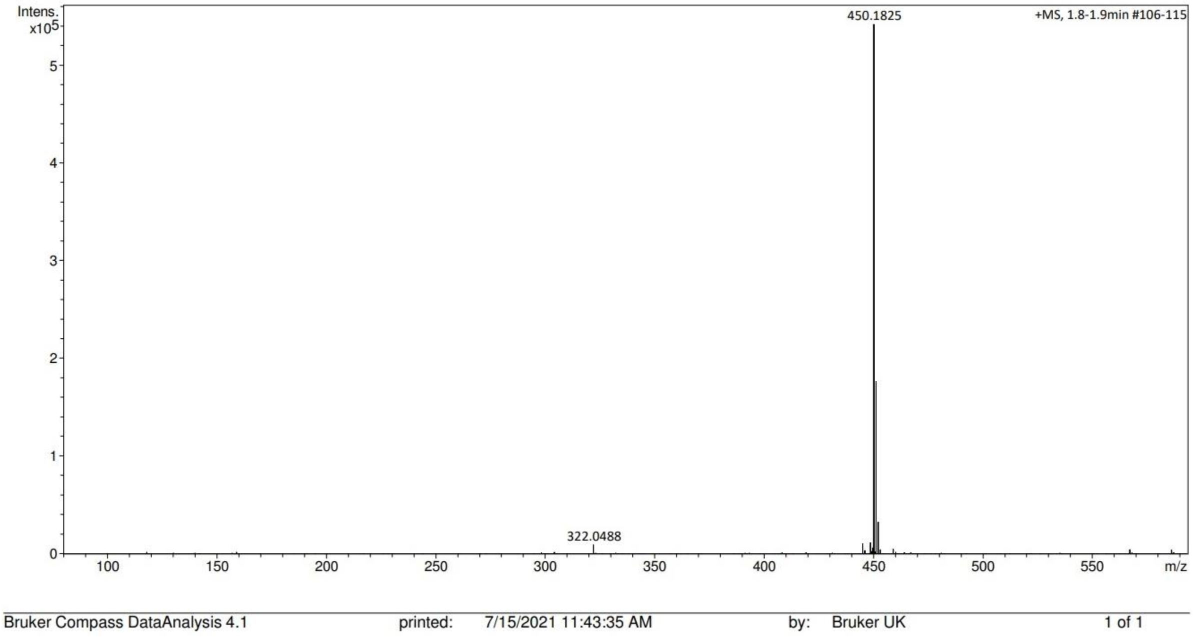

Figure S24. HR-MS of **DiKTa-DPA-OBuIm**.

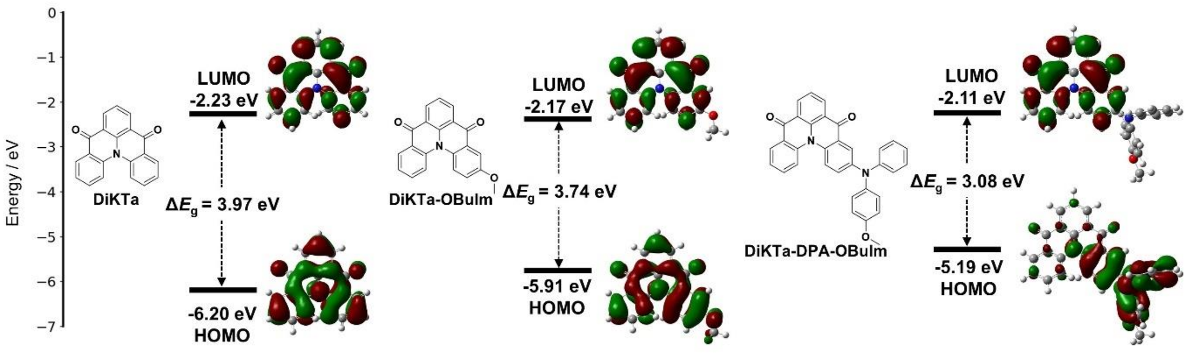

Figure S25. HOMO and LUMO electron density distribution and orbital energies of **DiKTa**, **DiKTa-OBuIm** and **DiKTa-DPA-OBuIm** calculated at PBE0/6-31G(d,p) in the gas phase, isovalue = 0.02.

Table S1. Calculated excited state energies and distance of charge transfer for each emitter at SCS-CC2/cc-pVDZ level in the gas phase.

**Compound**	**S_1 (D_{CT}) / eV (Å)**	f^a	**S_2 (D_{CT}) / eV (Å)**	f^a	**T_1 (D_{CT}) / eV (Å)**	**T_2 (D_{CT}) / eV (Å)**	**ΔE_{ST} / eV**
**DiKTa**	3.45 (1.45)	0.20	3.92 (0.63)	0.00	3.18 (1.00)	3.60 (0.34)	0.27
**DiKTa-OBulm**	3.31 (1.81)	0.20	3.81 (0.56)	0.00	3.04 (1.44)	3.54 (0.41)	0.27
**DiKTa-DPA-Obulum**	3.07 (3.34)	0.16	3.70 (3.34)	0.15	2.83 (2.79)	3.36 (1.66)	0.24

[a]Oscillator strength

Table S2. Energy levels extracted from the CV and DPV curves.

**Emitter**	E_{ox}^{a}	E_{red}^{a}	**HOMO^b /eV**	**LUMO^b / eV**	**$\Delta E_{H\text{-}L}^{c}$ / eV**
DiKTa-OBuIm	1.05	-1.67	-5.85	-3.13	2.72
DiKTa-DPA-OBuIm	0.44	-1.61	-5.24	-3.18	2.06

[a]In degassed MeCN with 0.1 M [nBu$_4$N]PF$_6$ as the supporting electrolyte and Fc/Fc$^+$ as the internal reference (0.38 V vs. SCE) [18]. [b]The HOMO and LUMO energies were determined using the relation $E_{HOMO/LUMO} = -(E_{ox} / E_{red} + 4.8)$ eV,[7] where E_{ox} and E_{red} are the anodic and cathodic peak potentials, respectively calculated from peak value of DPV referenced to Fc/Fc$^+$. [c]$E_{H\text{-}L} = |E_{HOMO}\text{-}E_{LUMO}|$.

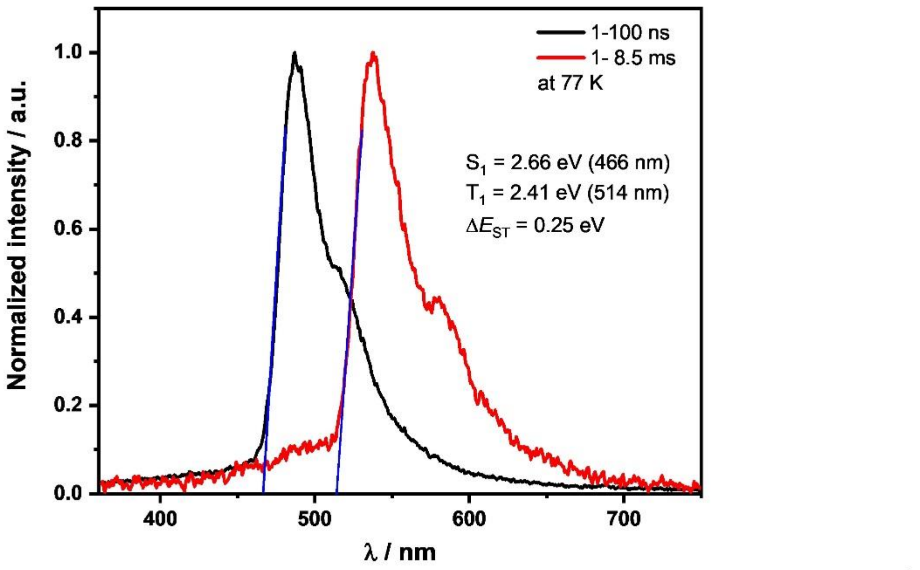

Figure S26. Steady-state fluorescence and phosphorescence spectra at 77 K of **DiKTa-OBuIm** measured in dil. 2Me-THF solutions. λ_{exc} = 343 nm.

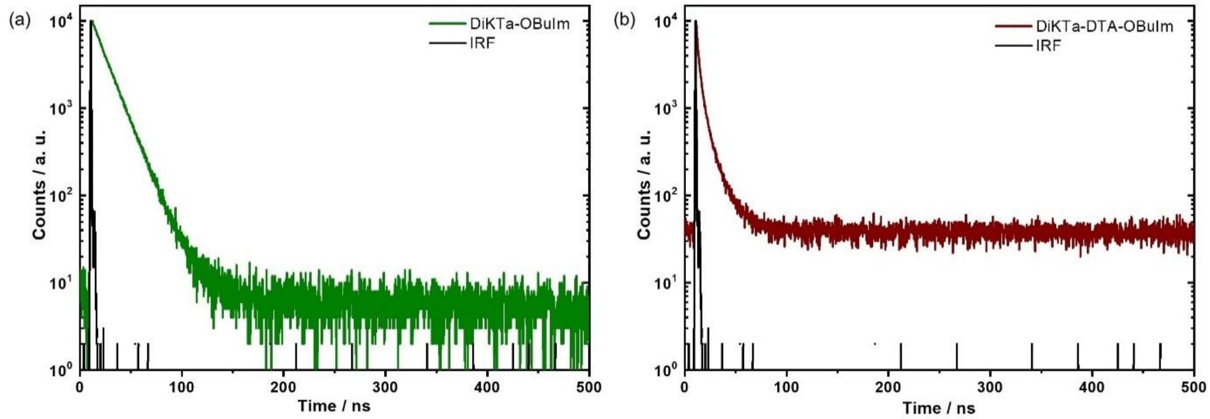

Figure S27. Decay of emission signal at rt under vac. of a) **DiKTa-OBuIm** and b) **DiKTa-DPA-OBuIm** in MeCN. λ_{exc} = 378 nm.

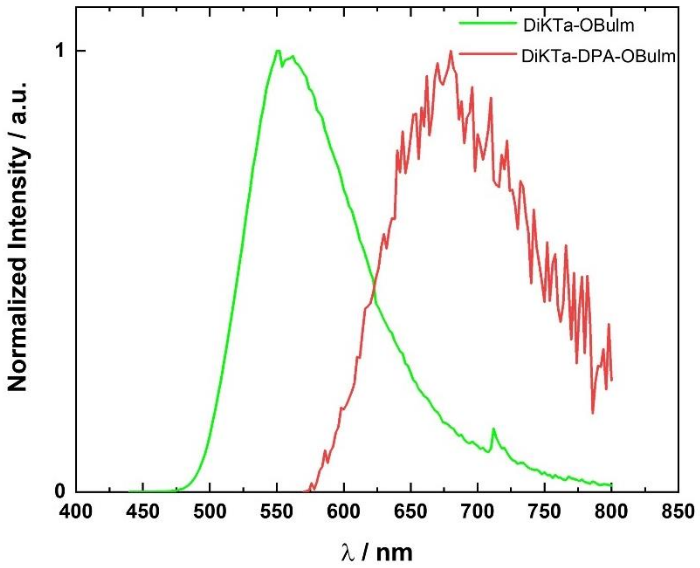

Figure S28. Steady-state emission spectrum of a) **DiKTa-OBuIm** $\lambda_{exc.}$ = 425 nm. and b) **DiKTa-DPA-OBuIm** in neat spin coated film measured at rt. $\lambda_{exc.}$ = 550 nm.

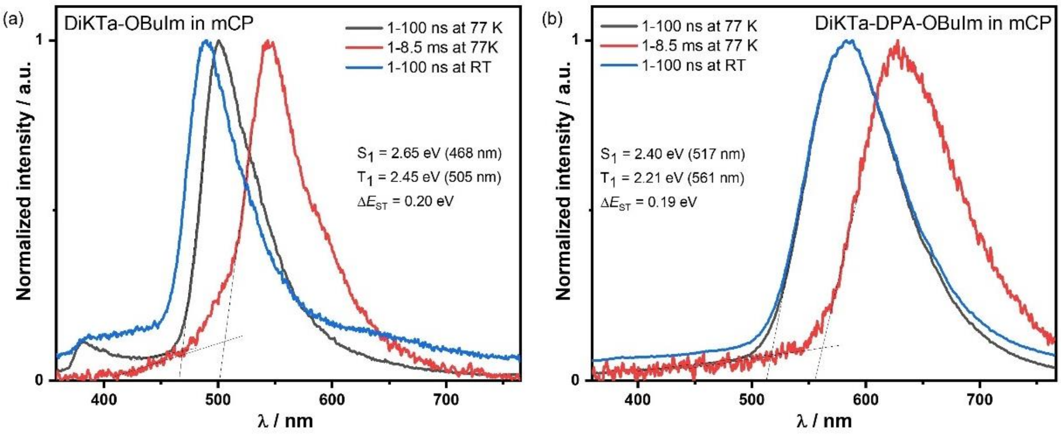

Figure S29. Fluorescence and phosphorescence spectra of a) **DiKTa-OBuIm** and b) **DiKTa-DPA-OBuIm** at 1 wt % doping in mCP spin-coated film measured at 77 K. $\lambda_{exc.}$ = 343 nm.

References

1. Ling, X.; Wilcox, C. S. *Chem. Eur. J.* **2019**, *25* (61), 14010-14014.
2. Shimizu, M.; Nakatani, M. *Eur. J. Org. Chem.* **2017**, *2017* (32), 4695-4702.
3. Wu, S.; Li, W.; Yoshida, K.; Hall, D.; Madayanad Suresh, S.; Sayner, T.; Gong, J.; Beljonne, D.; Olivier, Y.; Samuel, I. D. W.; Zysman-Colman, E. *ACS Appl. Mater. Interfaces* **2022**, *14* (19), 22341-22352.
4. Petersson, G. A.; Al-Laham, M. A. *J. Chem. Phys.* **1991**, *94* (9), 6081-6090.
5. Melhuish, W. H. *J. Phys. Chem.* **1961**, *65* (2), 229-235.
6. Tsuchiya, Y.; Diesing, S.; Bencheikh, F.; Wada, Y.; dos Santos, P. L.; Kaji, H.; Zysman-Colman, E.; Samuel, I. D. W.; Adachi, C. *J. Phys. Chem. A* **2021**, *125* (36), 8074-8089.
7. Cardona, C. M.; Li, W.; Kaifer, A. E.; Stockdale, D.; Bazan, G. C. *Adv. Mater.* **2011**, *23* (20), 2367-2371.

8. Frisch, M. J.; Trucks, G. W.; Schlegel, H. B.; Scuseria, G. E.; Robb, M. A.; Cheeseman, J. R.; Scalmani, G.; Barone, V.; Petersson, G. A.; Nakatsuji, H.; Li, X.; Caricato, M.; Marenich, A. V.; Bloino, J.; Janesko, B. G.; Gomperts, R.; Mennucci, B.; Hratchian, H. P.; Ortiz, J. V.; Izmaylov, A. F.; Sonnenberg, J. L.; Williams; Ding, F.; Lipparini, F.; Egidi, F.; Goings, J.; Peng, B.; Petrone, A.; Henderson, T.; Ranasinghe, D.; Zakrzewski, V. G.; Gao, J.; Rega, N.; Zheng, G.; Liang, W.; Hada, M.; Ehara, M.; Toyota, K.; Fukuda, R.; Hasegawa, J.; Ishida, M.; Nakajima, T.; Honda, Y.; Kitao, O.; Nakai, H.; Vreven, T.; Throssell, K.; Montgomery Jr., J. A.; Peralta, J. E.; Ogliaro, F.; Bearpark, M. J.; Heyd, J. J.; Brothers, E. N.; Kudin, K. N.; Staroverov, V. N.; Keith, T. A.; Kobayashi, R.; Normand, J.; Raghavachari, K.; Rendell, A. P.; Burant, J. C.; Iyengar, S. S.; Tomasi, J.; Cossi, M.; Millam, J. M.; Klene, M.; Adamo, C.; Cammi, R.; Ochterski, J. W.; Martin, R. L.; Morokuma, K.; Farkas, O.; Foresman, J. B.; Fox, D. J. *Gaussian 16 Rev. A.01*, Wallingford, CT, 2016.
9. **TURBOMOLE V7.4 2017, a development of University of Karlsruhe and Forschungszentrum Karlsruhe GmbH, 1989-2007, TURBOMOLE GmbH, since S105 2007, 1989-2007**.
10. Adamo, C.; Barone, V. *J. Chem. Phys.* **1999**, *110* (13), 6158-6170.
11. Petersson, G. A.; Al-Laham, M. A. *J. Chem. Phys.* **1991**, *94*, 6081-6090.
12. Thorn H. Dunning, J. *J. Chem. Phys.* **1989**, *90*, 1007-1023.
13. Hättig, C.; Weigend, F. *J. Chem. Phys.* **2000**, *113* (13), 5154-5161.
14. Hättig, C.; Hald, K. *Phys. Chem. Chem. Phys.* **2002**, *4* (11), 2111-2118, 10.1039/B110847F.
15. R Dennington; Keith, T.; Millam, J. *GaussView, Version 6.1.1*, KS: Semichem Inc.: Shawnee Mission, 2019.
16. Momma, K.; Izumi, F. *J. Appl. Crystallogr.* **2011**, *44* (6), 1272-1276.
17. Lu, T.; Chen, F. *J. Comput. Chem.* **2012**, *33* (5), 580-592.
18. Pavlishchuk, V. V.; Addison, A. W. *Inorg. Chim. Acta* **2000**, *298* (1), 97-102.
